# Gene-specific transcript buffering revealed by perturbation of coactivator complexes

**DOI:** 10.1126/sciadv.adr1492

**Published:** 2025-03-19

**Authors:** Faezeh Forouzanfar, David F. Moreno, Damien Plassard, Audrey Furst, Karen A. Oliveira, Bernardo Reina-San-Martin, László Tora, Nacho Molina, Manuel Mendoza

**Affiliations:** ^1^ Institut de Génétique et de Biologie Moléculaire et Cellulaire, Illkirch, France.; ^2^ Centre National de la Recherche Scientifique, UMR7104, Illkirch, France.; ^3^ Institut National de la Santé et de la Recherche Médicale, U964, Illkirch, France.; ^4^ Université de Strasbourg, Strasbourg, France.

## Abstract

Transcript buffering entails reciprocal modulation of mRNA synthesis and degradation to maintain stable RNA levels under varying cellular conditions. Current models depict a global connection between mRNA synthesis and degradation, but underlying mechanisms remain unclear. Here, we show that changes in RNA metabolism following depletion of TIP60/KAT5, the acetyltransferase subunit of the NuA4 transcriptional coactivator complex, reveal that transcript buffering occurs at a gene-specific level. By combining RNA sequencing of nuclear, cytoplasmic, and newly synthesized transcript fractions with biophysical modeling in mouse embryonic stem cells, we demonstrate that transcriptional changes caused by TIP60 depletion are offset by corresponding changes in RNA nuclear export and cytoplasmic stability, indicating gene-specific buffering. Disruption of the unrelated ATAC coactivator complex also causes gene-specific transcript buffering. We propose that cells dynamically adjust RNA splicing, export, and degradation in response to individual RNA synthesis alterations, thereby sustaining cellular homeostasis.

## Teaser

Altered synthesis of specific transcripts is counterbalanced by adjustments in their export and decay, preserving RNA homeostasis.

## INTRODUCTION

Eukaryotic gene expression involves a precise sequence of events, starting with the synthesis of mRNA precursors in the nucleus. These molecules are processed, spliced, and exported to the cytoplasm, where they undergo translation into proteins before ultimately undergoing degradation. Initially studied as distinct RNA metabolic processes, it is now clear that RNA synthesis and degradation are inherently linked. In particular, the development of methods to measure mRNA transcription and degradation rates uncovered connections between nuclear mRNA synthesis and cytoplasmic degradation. For instance, global reduction of transcription rates leads to a corresponding increase in mRNA stability in budding yeast (*1*–*3*) and in animal cells (*4*, *5*). Conversely, global inhibition of mRNA degradation is associated with decreased transcription rates (*2*, *6*, *7*). This phenomenon, termed “transcript buffering,” is thought to maintain mRNA concentration constant, which may be important in physiological contexts such as during changes in cell size (*8*–*10*).

The molecular mechanisms responsible for transcript buffering are unclear. Current models involve negative feedback on RNA polymerase II activity exerted by mRNA degradation factors (in yeast) or nuclear RNA (in animal cells) (*4*, *6*). These models aim to explain global buffering, wherein overall changes in mRNA synthesis are offset by overall changes in mRNA stability and vice versa. However, evidence suggests that transcript buffering may also operate at the gene-specific level. For instance, neurons deficient in the methyl-CpG-binding protein MeCP2, a transcriptional regulator, show alterations in both mRNA synthesis and mRNA half-life, with some changes compensating for others (*11*). It remains to be determined whether buffering occurs in a transcript-specific manner, the cellular contexts in which this mechanism operates, and how it responds to different transcriptional perturbations.

Dynamic control of gene expression involves collaboration among multiple protein types, including transcription factors and chromatin regulators such as the Tip60 complex, also known as NuA4. Tip60 is an evolutionarily conserved lysine acetyl-transferase (KAT) and chromatin remodeler complex with roles in transcription, DNA damage response, and intracellular signaling (*12*, *13*). Its KAT subunit TIP60, or KAT5, is essential for early mouse development. Knockdown of *Tip60* in mouse embryonic stem cells (ESCs) causes reduced proliferation, loss of pluripotency, and altered mRNA levels (*14*).

Given the strong correlation between transcription and acetylation of histones and chromatin-associated proteins (*15*), TIP60 would be expected to stimulate transcription. Unexpectedly, initial experiments in mouse ESCs (mESCs) revealed that although the level of some mRNAs is reduced after down-regulation of *Tip60*, most of the affected mRNAs actually become more abundant. This led to the proposal that TIP60 functions predominantly as a transcriptional repressor in ESCs (*14*, *16*). An alternative explanation, however, is that the observed increase of mRNA levels in TIP60-deficient cells could reflect posttranscriptional dysregulation. These initial studies measured RNA steady-state abundance, which depends on both RNA synthesis and degradation rates. Notably, the budding yeast homolog of TIP60 has been shown to promote both the synthesis and nuclear export of mRNA (*17*). In addition, TIP60 can bind mRNA (*18*, *19*), suggesting that TIP60 may also regulate gene expression posttranscriptionally. Therefore, the characterization of multiple steps in RNA metabolism is essential to understand the role of TIP60 in gene expression regulation.

Here, we investigated the effects of acute depletion of TIP60 on mRNA metabolism in mouse ESCs by deep sequencing of newly synthesized, nuclear and cytoplasmic RNA. We find that TIP60 acts mainly as a transcriptional activator, crucial for mRNA synthesis of its target genes. Only a small proportion of genes increased their transcription after TIP60 depletion, perhaps via indirect mechanisms. Strikingly, changes in transcription rates following TIP60 depletion are mirrored by opposite changes in RNA export and stability in both the nucleus and cytoplasm. Notably, the degree of this buffering effect corresponds precisely to the magnitude of transcriptional changes. Our findings indicate that in ESCs, transcript buffering operates at the gene-specific level, with important implications for our understanding of transcript buffering mechanisms.

## RESULTS

### Tip60 promotes transcription of its target genes

**Fig. 1.**
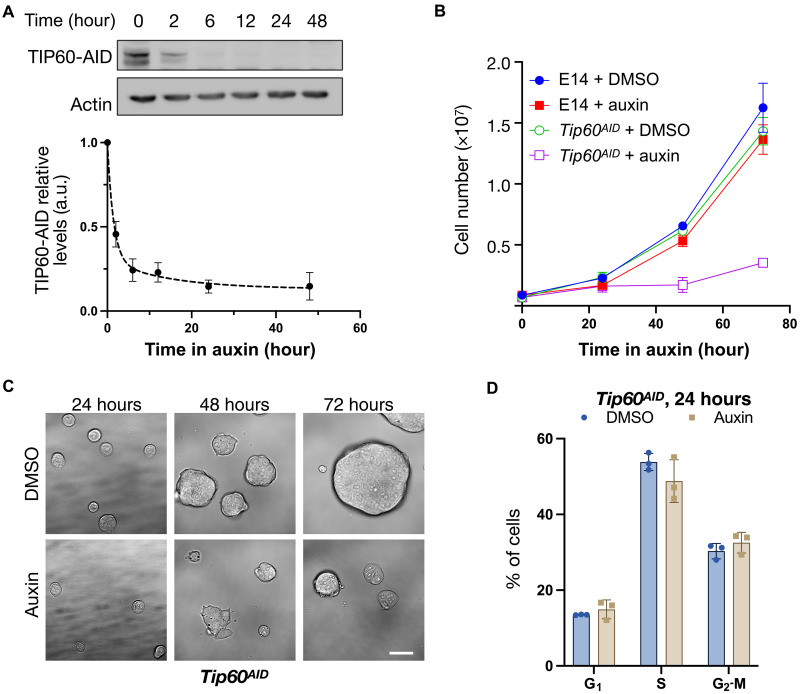
TIP60 is essential for mESCs proliferation. (**A**) *Tip60^AID^* cells were incubated for the indicated time with either DMSO or 1 mM auxin. TIP60 was detected by Western blotting using HRP-coupled streptavidin. Actin was used as a loading control. A representative Western blot and quantification of three independent replicates (mean and SEM) are shown. (**B**) Cell number (mean and SEM of *n* = 3 independent experiments) of the indicated cells treated with DMSO or 1 mM auxin at the indicated times. (**C**) Bright-field images of cells incubated with DMSO or 1 mM auxin for the indicated times. Scale bar, 40 μm. (**D**) Cell cycle distribution (mean and SD of three independent experiments) of *Tip60^AID^* cells treated with DMSO or auxin for 24 hours, determined by flow cytometry. Cells were grown in FCS + LIF + 2i. a.u., arbitrary unit.

We established a conditional degradation system designed for the rapid depletion of TIP60 upon auxin addition in mouse ESCs. Using CRISPR-Cas9 gene editing, we inserted sequences encoding an auxin-inducible degron (AID) and BioTag into both *Tip60* alleles of an ESC line expressing the rice F-box protein transport inhibitor response 1 (Tir1) (*20*), which drives auxin-dependent ubiquitination of AID proteins. We validated this cell line (*Tip60^AID^*) by polymerase chain reaction (PCR) (fig. S1) and showed that the TIP60-AID protein was efficiently eliminated within the first 6 hours of auxin treatment, with its loss sustained for >2 days (Fig. 1A).

To evaluate the impact of TIP60 depletion on cell survival, we examined *Tip60^AID^* cell proliferation during continuous auxin exposure for 24, 48, and 72 hours. Cells were cultured in medium containing leukemia inhibitory factor (LIF) and two inhibitors targeting mitogen-activated protein kinase (MEK)/extracellular signal–regulated kinase and glycogen synthase kinase 3 beta (GSK3b) pathways (LIF + 2i), which promote uniform expression of pluripotency genes (*21*). In this medium, *Tip60^AID^* cells proliferated at rates comparable to untagged TIP60-expressing (E14) cells. However, auxin addition specifically halted proliferation of *Tip60^AID^* cells after 24 hours (Fig. 1, B and C). Notably, *Tip60^AID^* cells cultured with LIF but without 2i, where pluripotency genes are expressed heterogeneously, proliferated more slowly than E14 cells. Auxin addition under these conditions also halted *Tip60^AID^* cell growth specifically (fig. S2). These results indicate that TIP60 is essential for ESC proliferation. Further, they suggest that AID tagging alone may partially impair TIP60 levels or function, at least in the absence of 2i. Supporting this, anti-TIP60 antibodies detected TIP60 in E14 but not in *Tip60^AID^* cells, likely reflecting reduced TIP60-AID levels or impaired antibody recognition (fig. S3A). We next measured mRNA levels of *Tip60* and of four putative TIP60 target genes (*Dub1*, *Zfp827*, *Efs*, and *Guca1A*) (*14*) by reverse transcription quantitative PCR (RT-qPCR). In the absence of auxin, *Tip60*, *Dub1*, *Guca1A*, and *Efs* levels were comparable in the two cell lines, whereas *Zfp827* levels were significantly altered in *Tip60^AID^* cells (fig. S3, B and C). Auxin treatment significantly altered expression of all four target genes, confirming TIP60 depletion (fig. S3C). In addition, pluripotency markers *Nanog* and *Oct4* were detected at comparable levels in both cell lines, and *Tip60^AID^* cells retained the ability to differentiate into neural precursors, albeit with slight delays. Differentiation was assessed by decreased *Nanog* and *Oct4* levels and increased expression of neuroectodermal markers *Sox1* and *Zfp521* (fig. S3D). We conclude that AID-tagged TIP60 is partially impaired but retains sufficient activity to support its key functions in gene expression and ESC proliferation and differentiation. The ability of 2i to complement TIP60-AID function may relate to its enhancement of *Nanog* expression (*21*), which was suggested to collaborate with TIP60 in maintaining ESC pluripotency (*14*). Auxin treatment depletes TIP60-AID efficiently, inhibiting proliferation.

To directly determine the role of TIP60 in RNA synthesis, we used transient transcriptome sequencing (TT-seq), which measures newly synthesized RNA (*22*). Following a 10-min incubation with 4-thiouridine (4sU) to label nascent RNA in Tip60^AID^ ESCs treated for 24 hours with dimethyl sulfoxide (DMSO) or auxin to deplete TIP60, nascent RNA was fragmented, purified, and quantified by Illumina sequencing. To ensure global normalization of TT-seq data, we added labeled RNA from *Drosophila*. TT-seq samples were enriched in intronic reads relative to RNA sequencing (RNA-seq) samples, consistent with reproducible and efficient isolation of newly synthesized transcripts (fig. S4). We conducted three independent sets of RNA-seq and TT-seq experiments using *Tip60^AID^* cells cultured in LIF + 2i medium, treated with auxin or DMSO for 24 hours. We selected this duration to ensure a complete Tip60 depletion in auxin-treated cells without detectable perturbation of cell growth or cell cycle progression (Fig. 1, A to D).

**Fig. 2.**
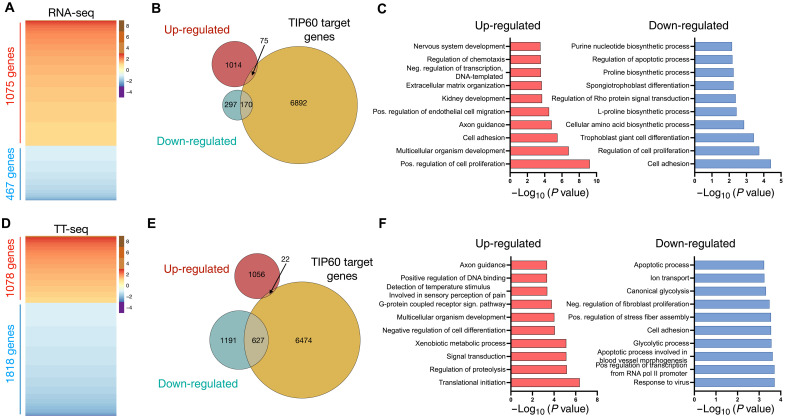
Role of TIP60 in mRNA synthesis. (**A**) Heatmaps of DEGs in *Tip60^AID^* cells treated with auxin versus DMSO for 24 hours assessed by RNA-seq. Genes in the heatmaps are sorted from the most up-regulated to the most down-regulated genes. (**B**) Venn diagrams designating the overlap between DEGs in (A) and TIP60-associated genes (*24*). (**C**) Top 10 GO terms (biological process) associated with DEGs in (A), ranked by *P* value. (**D** to **F**) Heatmaps (D), Venn diagrams (E), and GO analysis (F) of differentially transcribed genes assessed by TT-seq. Genes were considered significantly altered if their log2 (FC) was >1 or <−1 and their Benjamini-Hochberg adjusted *P* value < 0.05 (*n* = 3 independent experiments).

We compared data obtained with conventional RNA-seq with TT-seq for *Tip60^AID^* cells treated separately with auxin and DMSO. We classified RNAs as differentially expressed genes (DEGs) if they exhibited a greater than two fold change (FC) between the DMSO and auxin conditions, with a significance level of Benjamini-Hochberg adjusted *P* < 0.05. In RNA-seq experiments, we identified 1542 DEGs, with 70% displaying up-regulation (1075 mRNAs) and only 30% exhibiting down-regulation (467 mRNAs) (Fig. 2A). Down-regulated genes were significantly enriched for genes associated with TIP60 and the TIP60 complex component p400 (*P* < 0.01, hypergeometric test). Conversely, the up-regulated genes were less likely to interact with TIP60 directly (*P* = 1, hypergeometric test) (Fig. 2B and fig. S5) and included factors associated with multicellular development Gene Ontology (GO) terms (Fig. 2C). These results are consistent with previous *Tip60* small interfering RNA (siRNA) knockdown experiments that suggested that TIP60 represses differentiation genes (*14**,* *23*).

Intriguingly, our analysis of the TT-seq data showed that TIP60 depletion had a distinct effect on newly synthesized transcripts. More than 60% of DEGs (1818 of 2896 RNAs) showed a decrease in transcription efficiency, while <40% (1078 RNAs) showed an increase (Fig. 2D). Transcriptionally down-regulated genes were specifically enriched for genes associated with TIP60 and p400 (*P* < 0.01, hypergeometric test; Fig. 2E and fig. S5), while those exhibiting increased transcription included TIP60-independent developmentally regulated genes (Fig. 2F). We note that the overlap between downregulated genes with TIP60 targets is likely an underestimate because TIP60 complex target genes were identified by chromatin immunoprecipitation performed under standard ESC culture conditions without 2i (*18*, *24*). This suggests a large overlap between TIP60-bound genes under both 2i and 2i-free conditions.

In summary, TIP60 depletion predominantly causes reduced transcription of its target genes, suggesting that TIP60 likely functions as a transcriptional coactivator for these genes. This role is masked in total RNA-seq data, possibly due to compensatory changes in mRNA stability.

### Integration of TT-seq and Frac-seq data reveals gene-specific buffering

The above results suggest that the depletion of TIP60 affects more than one step in gene expression. To estimate how TIP60 depletion affects the rates at which RNAs flow from the nucleus to the cytoplasm and the relative stability of RNAs in these cellular compartments, we combined TT-seq with fractionation sequencing (Frac-seq) (*25*). Frac-seq allowed us to determine the FC of RNA isolated from the nuclear and cytoplasmic compartments in control versus TIP60-depleted cells, under the same conditions as the TT-seq experiments. We confirmed the absence of cross-contamination between nuclear and cytoplasmic fractions by RT-qPCR. Nuclear fractions were enriched in introns and nuclear long noncoding RNAs (lncRNAs), whereas mature mRNAs were predominantly found in the cytoplasmic fractions (fig. S6).

First, we used Frac-seq data to calculate the FC of RNA isolated from the nucleus and cytoplasm in control versus TIP60-depleted cells. Overall, TIP60 depletion did not cause a global accumulation of RNAs in either compartment. However, Frac-seq analysis detected significant changes in nuclear/cytoplasmic (N/C) ratio for specific RNA types, such as mRNAs encoding intronless genes (primarily canonical histones) and ribosomal proteins (fig. S7), as well as long (>50 kb) lncRNAs (fig. S8). Nonetheless, these changes were relatively small (less than twofold). Thus, TIP60 depletion has a modest impact on the N/C distribution of RNAs. Consistent with this, imaging of polyadenylated RNA by fluorescence in situ hybridization (FISH) showed that the overall mRNA (N/C) ratio was not affected by TIP60 depletion (fig. S9).

**Fig. 3.**
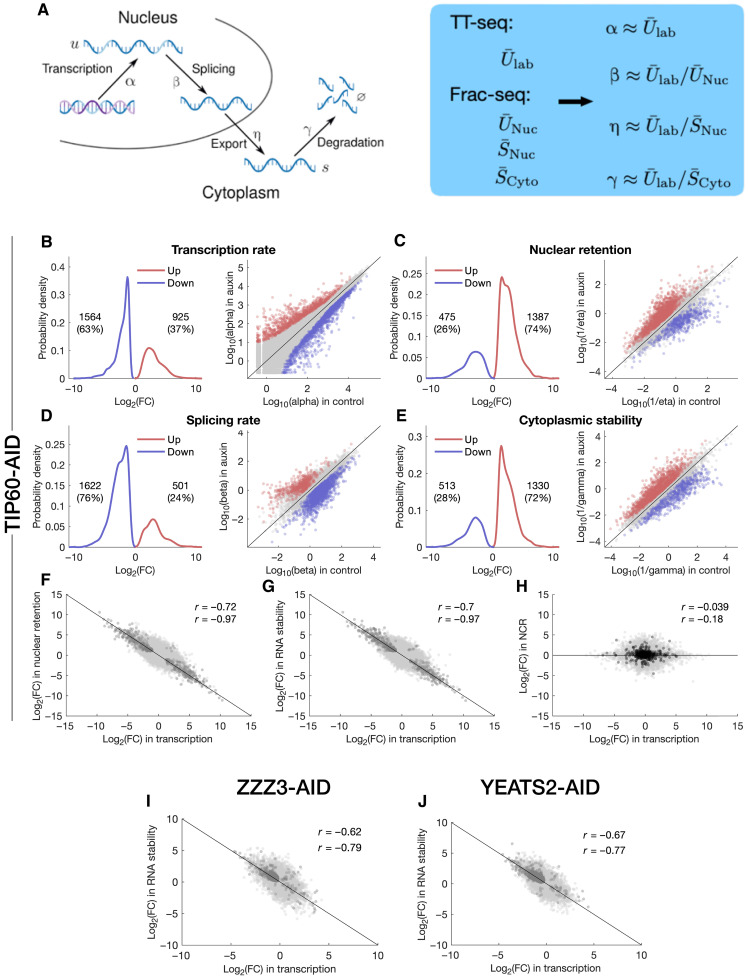
Integration of TT-seq and Frac-seq reveals gene-specific transcript buffering. (**A**) Schematic of mRNA metabolism, showing transcription (with rate α), splicing (β), export (η), and cytoplasmic degradation (γ). Rates are derived from TT-seq and Frac-seq data (blue box). Labels: ${\bar{U}}_{\text{lab}}$ (unspliced labeled RNA); ${\bar{U}}_{N}$, ${\bar{S}}_{N}$, and ${\bar{S}}_{C}$ (unspliced nuclear, spliced nuclear, and spliced cytoplasmic RNA). (**B** to **E**) Log_2_ FCs in transcription (B), splicing (C), nuclear retention (D), and cytoplasmic stability (E) upon TIP60 depletion. Significant genes (log_2_ FC > |1|, *P* < 0.01) are shown in red (up-regulated) and blue (down-regulated). Scatter plots show control versus auxin rates. Blue and red dots represent significant up- and down-regulated genes (*P* < 0.01, log_2_ FC > |1|). Gene numbers and relative percentages in each category are indicated. (**F** and **G**) Correlations between changes in transcription rate and changes in either nuclear retention (F) or cytoplasmic stability (G) upon TIP60 depletion. Dark dots represent genes with a significant FC (*P* < 0.01). Pearson correlation coefficients are displayed for all the genes (*r* = −0.72 and *r* = −0.7) and for significant genes (*r* = −0.97 and *r* = −0.97). (**H**) Log_2_ FC in transcription versus log_2_ FC in nuclear to cytoplasmic ratio (NCR). Black dots represent intronless genes. Pearson correlation coefficients are displayed for all the genes (*r* = −0.039) or intronless genes (*r* = −0.13). (**I** and **J**) Correlation between the log_2_ FC in transcription rate and the log_2_ FC in RNA stability after depletion of the ATAC subunit Zzz3 (I) or Yeats2 (J). Dark dots represent genes with a significant FC (*P* < 0.05). Pearson correlation coefficients are displayed for all the genes (*r* = −0.61 and *r* = −0.57) and for significant genes (*r* = −0.8 and *r* = −0.82).

Next, we integrated the TT-seq and Frac-seq datasets to fit a biophysical model that explicitly describes the following processes involved in RNA metabolism: transcription, splicing, nuclear export, and cytoplasmic degradation. This model extends the RNA velocity approach for analyzing spliced and unspliced RNA reads from bulk and single-cell RNA-seq experiments (*26*, *27*) to include the translocation between nuclear and cytoplasmic compartments. Furthermore, we assumed that the 24-hour auxin treatment was sufficiently long relative to the typical mRNA half-life, which is on the order of hours (*28*, *29*), allowing cells to attain a quasi-equilibrium state after perturbation. Consequently, we solved the model for the steady state, simplifying the fitting procedure (see Fig. 3A and Materials and Methods for extended details). Our approach allowed us to determine rates of RNA synthesis (α), splicing (β), nuclear export (η), and cytoplasmic stability (γ) of *Tip60^AID^* cells treated with auxin and DMSO. Note that consistent with reports in mouse and human cells (including mESCs) (*30*–*32*), we assumed that nuclear export is typically faster than nuclear degradation, and therefore, nuclear retention is dominated by the time mRNAs need to be translocated to the cytoplasm.

Our analyses show that upon TIP60 depletion, a greater number of RNAs exhibit reduced synthesis and splicing rates, while fewer RNAs display increased rates (Fig. 3, B and C). These trends are consistent with the role of TIP60 in promoting transcription and underscore the tight coupling between transcription and splicing (*33*, *34*). Conversely, we observed an opposing pattern for nuclear retention and cytoplasmic stability: More RNAs increase their nuclear and cytoplasmic half-lives in TIP60-depleted cells compared to control cells (Fig. 3, D and E). Strikingly, at the level of individual RNAs, the FCs in transcription were inversely proportional to the FCs in nuclear retention and cytoplasmic stability (Fig. 3, F and G). This led to maintenance of N/C ratios for most RNAs despite alterations in their synthesis rates (Fig. 3H). Notably, changes in nuclear retention and cytoplasmic stability were strongly correlated, suggesting coordination between changes in the two cellular compartments (fig. S10).

Gene-specific compensatory changes in RNA stability in response to altered RNA synthesis may be a specific response to TIP60 depletion. To test this, we compared changes in RNA abundance (measured with RNA-seq) to changes in RNA synthesis (measured with TT-seq) after inactivation of the Ada-two-A-containing (ATAC) complex, an unrelated transcriptional coactivator essential for ESC survival. The ATAC core subunits YEATS2 and ZZZ3, fused to AID, were depleted by addition of auxin for 24 hours (*20*). We found that under these conditions, changes in RNA synthesis due to ATAC perturbation also led to compensatory gene-specific changes in RNA stability and were distinct from those caused by TIP60 depletion (Fig. 3, I and J, and fig. S11). Together, these observations indicate a strong coupling among the transcription of single RNAs, their nuclear export/degradation, and their cytoplasmic decay, which we term gene-specific buffering.

The enhanced nuclear retention exhibited by RNAs with reduced synthesis rates in TIP60-depleted cells (shown in Fig. 3F) prompted us to investigate how transcription inhibition affects mRNA nuclear distribution in ESCs. We imaged polyadenylated RNA by FISH after a 2-hour treatment with the RNA polymerase II inhibitors triptolide and flavopiridol. This resulted in significant shifts in the subnuclear distribution of nuclear mRNA, which concentrated in nuclear speckles (fig. S12). Thus, reduced mRNA synthesis prompts the rapid redistribution of mRNA to nuclear speckles in ESCs.

### Buffering is less efficient for genes not bound to TIP60

**Fig. 4.**
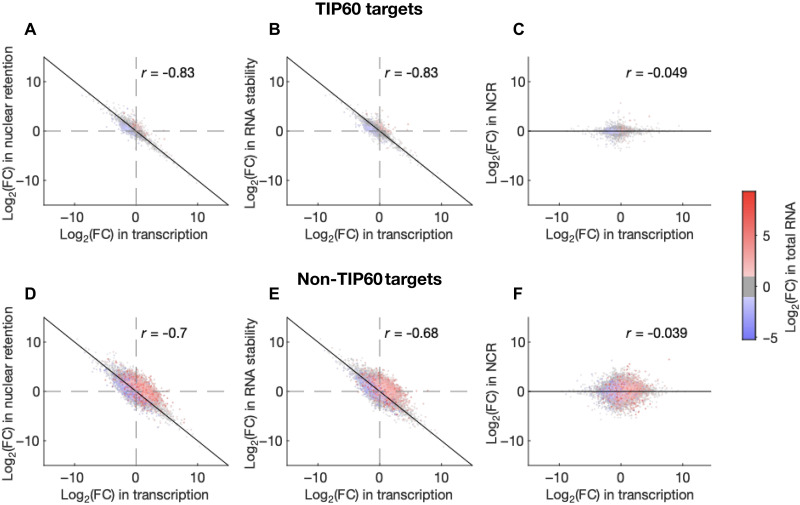
Buffering efficiency for Tip60 target and nontarget genes. Correlation between changes in transcription and nuclear retention (**A** and **D**), cytoplasmic stability (**B** and **E**), and N/C ratio (**C** and **F**) after TIP60 depletion as in Fig. 3 (F to H), for putative TIP60 target and nontarget genes. Changes in total RNA levels are color-coded as indicated.

Perfect buffering is expected to maintain constant RNA levels after perturbation of transcription. However, TIP60 depletion results in alterations in the levels of some RNAs (Fig. 2), suggesting that those genes are inefficiently buffered. To examine buffering efficiency, we overlaid RNA abundance data (derived from RNA-seq) onto plots correlating changes in RNA synthesis with changes in nuclear retention and cytoplasmic stability. Furthermore, we separately analyzed putative TIP60 target genes (Fig. 4, A to C) and nontarget genes (Fig. 4, D to F). Notably, RNA-seq data were not used to generate the biophysical model and therefore serve as an independent validation.

This analysis reveals that most RNAs with significant changes in transcription in TIP60-depleted cells (log_2_ FC > |1|, *P* < 0.01) are efficiently buffered, in both TIP60 target and nontarget genes (*r* = 0.99; gray dots in Fig. 4, A and B and D and E). In contrast, most changes in RNA abundance are associated with increases or decreases in transcription rates, which are incompletely compensated by corresponding changes in nuclear retention / cytoplasmic stability. RNAs exhibiting these changes are identifiable as, respectively, red or blue dots located away from the diagonal in Fig. 4 (A and B and D and E). Intriguingly, changes in RNA abundance are more frequent among putative non-TIP60 transcriptional targets. Specifically, 6% (418 of 6959) of putative TIP60 target genes are differentially expressed (greater than two FC), compared with 27% (6125 of 22713) of non-TIP60 targets. We consider this an underestimate, given that TIP60 targets were identified under 2i-free conditions; had these targets been chosen at random, we would not expect to see such a differential pattern. Thus, most of the gene expression changes following TIP60 depletion appear to stem from imperfect coordination between RNA synthesis and export/stability, predominantly affecting putative non-TIP60 target genes.

## DISCUSSION

The TIP60 KAT was initially identified as a transcriptional coactivator, able to acetylate various transcription factors and histone proteins (*13*, *35*, *36*). However, analysis of ES cells in which *Tip60* was down-regulated by siRNA suggested that TIP60 mostly acts as a transcriptional repressor (*14*, *16*). Here, we resolve this apparent contradiction by showing that while TIP60 actively promotes the transcription of a substantially larger set of genes compared to those it represses, this coactivator role is masked by compensatory posttranscriptional adjustments in RNA abundance. By exploring the mechanisms that explain this buffering effect, we reveal a gene-specific homeostasis mechanism that counterbalances changes in the synthesis of specific RNAs by adjusting their abundance in both the nucleus and cytoplasm. Thus, our findings have two major implications. First, we show that the major role of TIP60 in RNA synthesis in ESCs is to act as a transcriptional coactivator of specific target genes. Second, we find a discrepancy between changes in RNA abundance and synthesis rates that reveals strong coupling between the transcription of single RNAs, their nuclear export/degradation, and their cytoplasmic decay, a process we call gene-specific transcript buffering.

A role for TIP60 as a transcriptional coactivator is supported by the enrichment of TIP60 targets among genes exhibiting reduced transcription in TIP60-depleted cells, while genes displaying increased transcription did not display a direct interaction with TIP60. Therefore, the role of TIP60 in repressing transcription may be indirect. For instance, TIP60 may modulate the activity and/or expression of other transcriptional regulators or RNA binding proteins governing these genes. Supporting this possibility, TIP60 associates with transcriptional repressors including lysine deacetylases (*16*, *37*).

In cells depleted of TIP60, RNAs exhibiting reduced synthesis rates show increased retention in the nucleus and increased stability in the cytoplasm, while those with increased transcription display reduced residence in these compartments. Retention of mRNAs in the nucleus is associated with their recruitment to nuclear speckles (*38*, *39*), which may shield these mRNAs from degradation. Consistent with this hypothesis, we and others (*40*) observe the redistribution of mRNA to nuclear speckles after inhibition of transcription. Notably, the accumulation of mRNAs in nuclear speckles has also been observed after depletion of RNA export factors, including the nuclear pore component TPR (*38*, *39*).

**Fig. 5.**
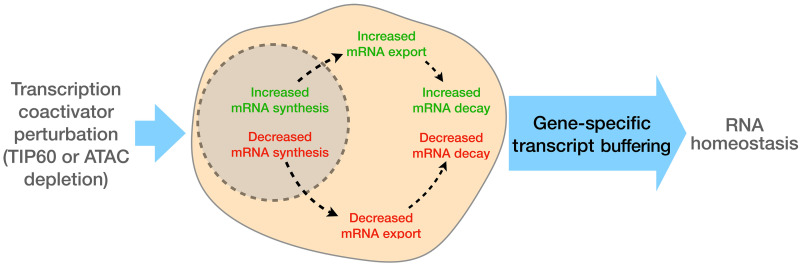
Gene-specific transcript buffering in response to transcription coactivator perturbations. This schematic illustrates the interplay between transcription coactivator perturbations (e.g., TIP60 or ATAC complex depletion) and RNA homeostasis. Perturbations lead to alterations in mRNA synthesis, which are counterbalanced by changes in mRNA export and cytoplasmic decay of specific transcripts, thereby maintaining RNA homeostasis.

Our findings are reminiscent of the known interdependence between global mRNA synthesis and degradation in yeast and animal cells, where inhibition of one of these processes leads to up-regulation of the other, a phenomenon termed “transcript buffering” (*8*, *9*). Transcript buffering is thought of as a global sensing mechanism responsive to changes in overall RNA synthesis. However, our data cannot be explained by models postulating that the activity of the transcriptional machinery regulates global RNA export and/or degradation activities (*8*, *9*). Instead, we see that buffering operates at the gene-specific level. Under our experimental conditions, both transcriptional repression and activation occur simultaneously, allowing us to capture dynamic and opposing adjustments to nuclear retention and cytoplasmic stability on a gene-by-gene basis. RNAs with reduced synthesis rates gain nuclear retention and cytoplasmic stability, while those with increased synthesis rates lose nuclear retention and cytoplasmic stability. Thus, as illustrated in Fig. 5, the buffering we detect does not simply respond to overall transcription rates but can be triggered by gene-specific perturbations, adjusting transcripts in both directions—up or down—at the individual gene level.

This gene-specific buffering may draw on similar mechanisms to those underlying global buffering. Yet, by demonstrating that gene-specific transcriptional changes elicit corresponding, finely tuned adjustments in nuclear retention and cytoplasmic stability, our findings broaden the concept of buffering beyond the global scale. They suggest that buffering mechanisms may operate at multiple levels of resolution: globally in response to uniform transcriptional stress, and locally to ensure individual genes maintain their appropriate expression levels under varied transcriptional environments. This conclusion has important implications for our understanding of buffering mechanisms.

The nature of the gene-specific buffering signal remains unclear. Our observation that transcriptional changes caused by perturbing two different coactivator complexes (TIP60 and ATAC) are efficiently buffered indicates that these coactivators themselves are likely not involved in the buffering mechanism. Intriguingly, promoter swap experiments in yeast suggest that mRNA stability is encoded within the promoter sequence, hinting at a connection between transcription and gene-specific buffering (*41*). Yet, the relevance of this phenomenon to gene-specific buffering in mammalian cells has not been directly investigated.

Our model assumes that nuclear RNA degradation is negligible compared to export, a view supported by data in mouse and human cells (*30*–*32*). In particular, only 579 transcripts show significant nuclear degradation in ESCs (*31*). We acknowledge that we cannot definitively exclude the possibility that TIP60 or ATAC depletion alters nuclear RNA stability. However, this seems unlikely, given that TIP60 depletion does not alter nuclear RNA levels as detected by polyadenylate [poly(A)] FISH and that our Frac-seq data show no evidence of global changes in nuclear RNA retention. Consequently, we consider any impact of these perturbations on bulk nuclear RNA stability to be minimal. Furthermore, transcript-specific differences in nuclear degradation alone cannot explain our observations. While increased transcription could coincide with elevated nuclear degradation, thereby preserving stable nuclear RNA levels for transcripts with heightened transcription, it cannot explain stable levels of transcripts whose synthesis rate is reduced because their degradation is already negligible. Thus, reduced nuclear export would still be required to maintain stable nuclear levels of these transcripts, which constitute the majority under TIP60 and ATAC depletion. In summary, the most parsimonious explanation of our data is that changes in nuclear export, rather than in nuclear degradation, predominantly underlie gene-specific buffering at the nuclear level.

Notably, our modeling indicates that changes in nuclear retention and cytoplasmic degradation are coupled to each other. We speculate that buffering mechanisms involve the direct coupling of gene-specific RNA synthesis rates with their nuclear export and cytoplasmic degradation rates. This coupling might be mediated by RNA “marks” that adjust RNA export and stability in proportion with RNA synthesis rates, possibly through association with RNA binding proteins or specific RNA modifications. An example of a mark deposited proportionally to RNA synthesis rate is N6-methyladenosine (m6A), which is slightly more abundant in slow-transcribing mRNAs. However, this modification drives mRNA instability (*42*–*44*), arguing against its direct involvement in gene-specific buffering. The impact of other synthesis rate-dependent RNA marks on its nuclear export and stability, and their potential involvement in buffering mechanisms, remains to be investigated. In addition, exploring how buffering mechanisms are modulated during critical cellular transitions, such as ESC differentiation, presents a fascinating area for future research.

## MATERIALS AND METHODS

### Cell culture

ESCs were grown on 0.1% gelatinized (Sigma-Aldrich, catalog no. G1890) tissue-culture plates in Dulbecco’s modified Eagle’s medium [glucose (4.5 g/liter)] with GlutaMAX-I supplemented with ES-tested 15% fetal calf serum (FCS; Thermo Fisher Scientific, catalog no. 10270-106), 0.1% β-mercaptoethanol (Thermo Fisher Scientific, catalog no. 31350-010), penicillin (100 U/ml), streptomycin (100 μg/ml; Thermo Fisher Scientific, catalog no. 15140-122), 0.1 mM non-essential amino acids (Thermo Fisher Scientific, catalog no. 11140-035), and LIF (1500 U/ml; produced in-house). A 3 μM CHIR99021 (Axon Medchem, catalog no. 1386) and 1 μM PD0325901 (Axon Medchem, catalog no. 1408) (2i) were added freshly to the medium, which was replaced every 24 hours. Cells were kept in G418 (400 μg/ml) for Tir1 selection. Auxin (3-indoleacetic acid, Sigma-Aldrich, I2886) was used at 1 mM for TIP60 depletion, and at 500 μM for YEATS2 and ZZZ3 depletion. ESC numbers were assessed using a Countess II Automated Cell Counter (Invitrogen). *Drosophila melanogaster* Schneider S2 cells (CRL-1993, ATCC) were grown in Schneider’s *Drosophila* medium (Thermo Fisher Scientific, catalog no. 21720-024) containing 10% FCS (heat inactivated) (Sigma-Aldrich, catalog no. F7524) and 0.5% penicillin and streptomycin at 27°C. For mESC differentiation, cells were plated in N2B27 medium for the number of days indicated, as described in (*16*).

### Plasmid construction

The plasmid expressing two guide RNAs (gRNAs; sequence: last exon, 5′-ACTGGAGCAAGAGAGGAAAG-3′; 3′ untranslated region, 5′-CACGAGAGCTGGCCGAACCA-3′) target the 3′ end of the endogenous the *Tip60* (*Kat5*) locus and coexpress the high-fidelity Cas9 nuclease (Cas9-HF) (*45*). The homologous recombination (HR) template contains homology arms of approximately 800 base pair (bp) surrounding the AID-FLAG-BioTag-P2A-EGFP construct. This allows TIP60 detection via the Flag tag and BirA-dependent biotinylation of the biotin acceptor peptide, as well as identification of positive clones via fluorescence of free enhanced green fluorescent protein (EGFP) owing to the P2A self-cleaving peptide. All gRNA/Cas9-HF and HR plasmids were generated through Golden Gate cloning (*46*).

### Generation of Tip60 AID cell line

Tir1-BirA mouse ES cells (PGKpr:Tir1-HA-IRES-3xHA-BirA-SV40pr:NeoR) were produced as previously described (*20*) and tested negative for mycoplasma contamination. CRISPR-Cas9 was used to generate mouse ES cells with TIP60 endogenously tagged with AID. Tir1-BirA mouse ES cells at a confluency of 70 to 80% were transfected with the plasmid constructs using Lipofectamine 2000 (Thermo Fisher Scientific, catalog no. 11668019) following the manufacturer’s instructions. The donor plasmid (Tip60-AID-Flag-BioTag-P2A-EGFP) was linearized using a restriction enzyme before transfection and transfected together with a Cas9-containing transient plasmid in the Tir1-BirA–expressing cell line. Cells were sorted by fluorescence-activated cell sorting by GFP expression 2 to 3 days after transfection. Three to five 96-well plates were seeded with one fluorescent cell per well using the BD FACSAria II (BD Biosciences), following the manufacturer’s instructions. The primer pairs that were used for genotyping were the following: forward, 5′-GAGCCCCCTGTCC-TTTCCTATTATG--3′; reverse, 5′-AAGGGAGATGGTAGTTTGGGGTGAGGGCAGTAGC-3′.

### Cell cycle analysis

Cell cycle profiles were determined by labeling of propidium iodide (PI) and followed by flow cytometry analysis. Cells were fixed with 70% ethanol and 30% phosphate-buffered saline (PBS) at −20°C for at least 30 min. After being washed once with PBS, cells were treated with ribonuclease A (Sigma-Aldrich, catalog no. R6513, 50 μg/ml in PBS) for 30 to 60 min. Cells were incubated with PI (25 μg/ml in PBS; Sigma-Aldrich, catalog no. P4864) at 37°C for 20 min. The samples were analyzed by flow cytometry using a MACSQuant flow cytometer (Miltenyi Biotec Inc.); data were visualized and quantified using FlowJo (Becton Dickinson).

### Whole-cell protein extraction

Cells were harvested and washed twice with 1x PBS. The cell pellet was resuspended in 1 volume of whole-cell extract buffer [50 mM tris-HCl (pH 7.9), 25% glycerol, 0.2 mM EDTA, 0.5 mM dithiothreitol (DTT), 5 mM MgCl_2_, 600 mM KCl, 0.5% NP-40, and 1x protein inhibitor cocktail] and incubated for 10 min at 4°C. The salt concentration was neutralized by adding 3 volumes of IP0 buffer [25 mM tris-HCl (pH 7.9), 5% glycerol, 5 mM MgCl_2_, 0.1% NP-40, 1 mM DTT, and 1x protein inhibitor cocktail] and incubated for 10 min at 4°C. After centrifugation at 12,000*g* for 10 min at 4°C, supernatants containing proteins were collected and stored at −80°C. Protein concentrations were determined using the Bradford method, using a SmartSpec 3000 spectrophotometer (Bio-Rad).

### Western blot analysis

Proteins boiled in Laemmli buffer were separated on NuPAGE 4 to 12% gradient bis-tris SDS–polyacrylamide gel electrophoresis gels, transferred onto a Hybond-C nitrocellulose membrane (GE Healthcare), and then blocked with TBST containing 5% (w/v) non-fat milk (TBSTM), for 30 min. Membranes were incubated overnight in primary antibodies diluted 1:1000 in TBS-Tween containing 1% (w/v) non-fat milk [or 1% bovine serum albumin (BSA) for streptavidin-horseradish peroxidase (HRP)] at 4°C. Membranes were washed three times with TBST, incubated in HRP-conjugated anti–immunoglobulin G (IgG) secondary antibodies (Cell Signaling Technology) in TBSTM at room temperature, followed by a further three washes with TBST. The membranes were developed using the Pierce ECL Western Blotting Substrate (SuperSignal West Pico PLUS Chemiluminescent Substrate) and visualized using a ChemiDoc Imaging System (Bio-Rad). Streptavidin protein fused to HRP (Thermo Fisher Scientific, 21126) was used to detect the TIP60-AID-Flag-BioTag fusion protein. Actin was detected with anti–β-actin (A5316, Sigma-Aldrich) antibodies. TIP60 and vinculin were detected with antibodies sc-166323 at 1:100 in 5% milk and sc-73614 1:10,000 in 5% milk + TBST.

### Total RNA extraction

Total RNA extraction was performed using TRI Reagent (Molecular Research Center Inc., catalog no. TR 188), following the manufacturer’s instructions. Deoxyribonuclease (DNase) I treatment was performed to prevent genomic DNA contamination using the TURBO DNA-free Kit (Thermo Fisher Scientific, catalog no. AM1907) per manufacturer’s instructions.

### 4sU metabolic labeling

Cells (5 × 10^7^) from three independent cultures were treated with auxin or DMSO. Twenty-four hours after auxin treatment, the nucleoside analog 4sU (Glentham Life Sciences, catalog no. GN6085) was added to a final concentration of 500 µM for a 10-min pulse at 37°C and 5% CO_2_. After labeling, cells were washed with ice-cold 1x PBS and immediately lysed using TRI Reagent (Molecular Research Center Inc., catalog no. TR 188).

### Purification of newly synthesized RNA

Newly synthesized RNAs were purified as previously described in detail (*22*, *47*, *48*). Briefly, 4sU-labeled total RNA of spike-in cells (*D. melanogaster*) was added to 250 μg of labeled total RNA from mouse ESCs in a ratio 1:10 before newly synthesized RNA purification. The RNA was precipitated and resuspended in 130 μl of RNase-free water (Sigma-Aldrich, catalog no. 95284) and sonicated on a E220 Focused-ultrasonicator (Covaris) using the following settings: 1% duty factor, 100 W, 200 cycles per burst, and 80 s, to obtain fragment size range from 10 kb to 200 bp. For purification, the fragmented total RNA was incubated for 10 min at 60°C and immediately chilled on ice for 2 min to open secondary RNA structures. Biotinylation was performed in labeling buffer [10 mM Hepes-KOH (pH 7.5) and 1 mM EDTA] and biotin-HPDP (0.2 mg/ml; Thermo Fisher Scientific, catalog no. 21341) for 3 hours at room temperature at 24°C in the dark and with gentle agitation. Unbound biotin-HPDP was removed by adding an equal volume of chloroform/isoamyl alcohol (24:1) at 16,000*g* for 5 min at 4°C. RNA was precipitated at 20,000*g* for 20 min with a 1:10 volume of 5 M NaCl and an equal volume of 100% isopropanol. The pellet was washed with an equal volume of 75% ethanol and precipitated again at 20,000*g* for 10 min. The pellet was resuspended in 100 μl of RNase-free water. Biotinylation and purification of 4sU-labeled RNAs was performed as described (*49*, *50*). Biotinylated RNA was captured using 100 μl of streptavidin-coated μMACS magnetic beads (Miltenyi Biotec, catalog no. 130-074-101) for 90 min at 24°C under gentle agitation. The μMACS columns (Miltenyi Biotec, catalog no. 130-074-101) were placed on a MACS MultiStand (Miltenyi Biotec) and equilibrated with washing buffer [100 mM tris-HCl (pH 7.5), 10 mM EDTA, 1 M NaCl, and 0.1% Tween 20] twice on the columns before adding the samples. The columns were then washed once with 600 μl, 700 μl, 800 μl, 900 μl, and 1 ml with washing buffer. Flow-through was collected for recovery of unlabeled preexisting RNA. RNA-4sU was eluted with two washes of 100 μl of freshly prepared 100 mM DTT. RT was performed with 1 μg of total RNA and using 0.2 μg of random hexamer primers (Thermo Fisher Scientific, catalog no. SO142) and 200 U of SuperScript IV Reverse Transcriptase (Thermo Fisher Scientific, catalog no. 18090050) following the manufacturer’s instructions.

### Quantitative PCR

Real-time quantitative PCR reactions were performed using a LightCycler 480 system (Roche) with SYBR Green 2× PCR Master Mix I (Roche, catalog no. 04887352001) and 1 μM forward and reverse primer, respectively. The primer pairs used for qPCR are listed in table S1. Relative gene expression was calculated on the basis of the obtained threshold values using the 2^−(ΔΔCT)^ method (*51*).

### RNA Frac-seq

Nuclear and cytoplasmic RNAs were purified as previously described in detail (*25*). Optimizing cell fractionation techniques for ESCs enabled the isolation of nuclear and cytoplasmic fractions from control and TIP60-depleted cells, followed by the purification and sequencing of their respective RNA content. Briefly, cells growing in three 150-mm plates at 60 to 80% confluency were harvested with trypsin followed by centrifugation at 12,000 rpm for 5 min, and then the supernatant was discarded and the cell pellet washed three times with 1x PBS. Subsequently, 10% of cell volume was subjected as the total RNA fraction, and the remaining (90%) was resuspended in 500 μl of φ buffer [150 mM potassium acetate, 5 mM magnesium acetate, 20 mM Hepes (pH 7.4), 1 mM sodium fluoride, 1 mM sodium orthovanadate, 25× protease inhibitor cocktail (Roche), 1:1000 dilution of SUPERase In RNase (Invitrogen), and 0.1% diethylpyrocarbonate]. Then, 500 μl of φ buffer containing 1% Triton X-100 (Thermo Fisher Scientific) and 0.2% sodium deoxycholate was gently added to the resuspended cells and incubated for 3 min on ice. After that, the cell sample was centrifuged at 12,000 rpm for 5 min. Last, cytoplasmic RNAs were extracted from the supernatant, and the nuclear RNAs were extracted from the pellet using TRI Reagent (Molecular Research Center Inc., catalog no. TR 188) as per the manufacturer’s instructions. The same RNA extraction procedure was performed to extract RNA from the “total” fraction. DNase I treatment was performed to prevent genomic DNA contamination using the TURBO DNA-free Kit (Thermo Fisher Scientific, catalog no. AM1907), following the manufacturer’s instructions.

### Library preparation and sequencing

Total RNA-seq libraries were generated from 500 ng of total RNA. Before cDNA synthesis, cytoplasmic and mitochondrial ribosomal RNA (rRNA) were removed using a biotin-streptavidin magnetic bead-based procedure with the riboPOOL Kit targeting HMR ribosomal rRNA (siTOOLs Biotech, Planegg/Martinsried, DE) according to the manufacturer’s instructions. Total RNA-seq libraries were then generated using TruSeq Stranded mRNA Library Prep kit and TruSeq RNA Single Indexes Kits A and B (Illumina, San Diego, CA) omitting the poly(A) selection step and starting from the fragmentation step. Total RNA-seq libraries were generated from 50 ng of total RNA for the TT-seq experiment and from 600 ng of total RNA for the Frac-seq experiment using Illumina Stranded Total RNA Prep, Ligation with Ribo-Zero Plus kit and IDT for Illumina RNA UD Indexes, Ligation (Illumina, San Diego, USA), according to the manufacturer’s instructions. Abundant rRNAs were depleted by hybridization to specific DNA probes and enzymatic digestion. Briefly, for these three experiments, the depleted RNA was fragmented into small pieces using divalent cations at 94°C for 2 min. Cleaved RNA fragments were then copied into first strand cDNA using reverse transcriptase and random primers followed by second strand cDNA synthesis using DNA polymerase I and RNase H. Strand specificity was achieved by replacing 3′-deoxythymidine 5′-triphosphate with deoxyuridine triphosphate during second strand synthesis. The double-stranded cDNA fragments were blunted using T4 DNA polymerase, Klenow DNA polymerase, and T4 PNK. A single “A” nucleotide was added to the 3′ ends of the blunt DNA fragments using a Klenow fragment (3′ to 5′exo minus) enzyme. The cDNA fragments were ligated to double-stranded adapters using T4 DNA Ligase. The ligated products were enriched by PCR amplification (30 s at 98 °C; [10 s at 98°C, 30 s at 60°C, and 30 s at 72°C] × 12 cycles (13 cycles for TT-seq); 5 min at 72°C). Surplus PCR primers were further removed by purification using AMPure XP beads (SPRIselect beads for TT-seq and Frac-seq) (Beckman Coulter, Villepinte, France), and the final cDNA libraries were checked for quality and quantified using capillary electrophoresis. All the libraries were sequenced with 2 × 100 base pairs on an Illumina HiSeq 4000 sequencer or on an Illumina NextSeq 2000 sequencer for the Frac-seq experiment. Image analysis and base calling were carried out using RTA v.2.7.3 and bcl2fastq v.2.17.1.14.

### Sequence analysis total RNA-seq, TT-seq, and Frac-seq

Reads were preprocessed using CUTADAPT v.1.10 (*52*) to remove adaptors and low-quality sequences and reads shorter than 40 bp. rRNA sequences were removed for further analysis. Reads were mapped onto the mm10 assembly of the *Mus musculus* genome using STAR v.2.5.3a (*53*). For TT-seq data, due to the spike-in, reads were mapped onto a hybrid genome composed of *M. musculus* and *D. melanogaster*. Gene expression was quantified from uniquely aligned reads using HTSeq-count v.0.6.1p1 (*54*) with annotations from Ensembl release 102 and union mode (and “-t gene” for TT-seq data in order to take into account reads aligned onto introns). Only non-ambiguously assigned reads have been retained for further analyses. Comparisons of interest have been performed using R 3.3.2 with DEseq2 version 1.16.1 (*55*). More precisely, read counts were normalized from the estimated size factors using the median-of-ratios method and a Wald test was used to estimate the *P* values. *P* values were then adjusted for multiple testing with the Benjamini and Hochberg method (*56*). For TT-seq data, size factors were estimated using *Drosophila* spike-in. To determine whether a category of mRNA was more affected by the depletion of Tip60, genes with a mean Fragments Per Kilobase of transcript per Million mapped reads (FPKM) > 1 were considered and genes from the mitochondrial genome were excluded. For protein coding genes or lincRNA, bins were created according to the median pre-mRNA length (<10, 10 to 20, 20 to 30, 30 to 40, 40 to 50, and >50 kb) or according to the number of exons (1, 2, 3, 4, 5, 6, 7, and 8 for protein coding genes and 1, 2, 3, 4, and 5 for lncRNA). DESeq2 log_2_ FCs (with FC shrinkage) from auxin-treated versus control comparison were compared between bins using Mann-Whitney–Wilcoxon tests with Benjamini-Hochberg adjustment for multiple comparisons. For Frac-seq data, the difference of DESeq2 log_2_ FCs (log_2_(nuclear/cytoplasmic)_auxin-treated_ − log_2_(nuclear/cytoplasmic)_Control_) was used for the comparisons. Genes having a length < 10 kb (or one exon) were compared to genes from other length bins (or other exon bins). The same approach was used to determine whether the canonical or non-canonical histones were affected by the depletion of TIP60.

### ChIP-seq analysis

FASTQ files were retrieved from GEO for p400IP_WT and IgG (*14*). After read mapping onto mm10 mouse genome using Bowtie2 v.2.4.5, BAM files were converted into BED files using BEDTools v.2.30.0. Blacklisted regions, available at https://github.com/Boyle-Lab/Blacklist/tree/master/lists, were removed from the BED files. Peak calling was performed using MACS2 v.2.7.1, and IgG was used as control. Peak annotation was carried out using annotatePeaks.pl program from HOMER v.4.11, with Ensembl version 102 as annotation file.

### Mathematical modeling of RNA metabolism

To integrate TT-seq and Frac-seq data, we designed a biophysical model to describe mRNA accumulation in both the nucleus and the cytoplasm, extending the previous RNA velocity approach (*26*, *27*). In particular, we assume that unspliced nuclear mRNA ($u_{N}$) is synthesized with a constant transcription rate α and spliced out with a constant splicing rate β. In turn, spliced nuclear mRNA ($s_{N}$) is translocated into the cytoplasm with a constant export rate η. Last, spliced cytoplasmic mRNA ($s_{c}$) is degraded with a constant degradation rate γ. Consequently, mRNA accumulation dynamics in the different compartments is governed by the following system of ordinary differential equations

$$\frac{du_{N}}{\text{dt}}=\alpha -\beta u_{N}$$ (1)

$$\frac{ds_{N}}{\mathrm{dt}}=\beta u_{N}-\eta s_{N}$$ (2)

$$\frac{ds_{C}}{\mathrm{dt}}=\eta s_{N}-\gamma s_{C}$$ (3)

Note that the effective rates α, β, η, and γ are considered gene specific and characterize the effective speed at which complex multisteps processes of mRNA metabolism occur. Furthermore, we assume that mRNA export is typically faster than nuclear degradation (*57*), and therefore, the nuclear retention of spliced mRNA is mainly governed by mRNA translocation.

The typical mRNA half-life has been reported to be in the order of 9 hours (*28*). Therefore, we assumed that cells reached a quasi-equilibrium state after the 24-hour treatment with auxin. Consequently, we solved the model for the steady state by setting the right-hand side of the equations above to zero, yielding the following result

$$u_{N}=\frac{\alpha }{\beta }$$ (4)

$$s_{N}=\frac{\alpha }{\eta }$$ (5)

$$s_{C}=\frac{\alpha }{\gamma }$$ (6)

By mapping intronic and exonic reads from the Frac-seq experiments, we were able to obtain estimates for the levels of nuclear unspliced and spliced mRNA, as well as cytoplasmic spliced mRNA averaged over the replica, denoted as ${\bar{U}}_{N}$, ${\bar{S}}_{N}$, and ${\bar{S}}_{C}$. In addition, from TT-seq experiments, we estimated the labeled unspliced mRNA from TT-seq averaged over replica, ${\bar{U}}_{\text{lab}}$. Plugging these quantities into Eqs. 4 to 6 and assuming that labeled RNA is a good readout of transcription rate, we obtain the following gene-specific estimates for the key rates of RNA metabolism up to a scaling factor

$$\alpha \approx {\bar{U}}_{\text{lab}}$$ (7)

$$\beta \approx {\bar{U}}_{\text{lab}}/{\bar{U}}_{N}$$ (8)

$$\eta \approx {\bar{U}}_{\text{lab}}/{\bar{S}}_{N}$$ (9)

$$\gamma \approx {\bar{U}}_{\mathrm{lab}}/{\bar{S}}_{C}$$ (10)

Last, we calculated errors for each rate estimate based on the SEs of unspliced and spliced mRNA levels by applying error propagation.

For depletion of the ATAC subunits Zzz3 and Yeats2, TT-seq data were obtained from (*20*), and RNA-seq was performed under the same conditions. In the absence of Frac-seq data, RNA degradation rates were estimated from Eq. 10 in which total spliced RNA levels in whole cells were used instead of cytoplasmic levels. Gene-specific rates are provided in table S2 [wild type (WT) and TIP60 depletion] and table S3 (WT, YEATS2, and ZZZ3 depletion).

To facilitate the inspection and subsequent analysis of buffered gene products, we defined nuclear, cytoplasmic, and total buffering indices (*BI*) as

$$BI_{\text{Nuc}}=|\text{log}2\text{FC}\left(\alpha \right)|-|\text{log}2\text{FC}\left(S_{N}\right)|$$ (11)

$$BI_{\text{Cyt}}=|\text{log}2\text{FC}\left(\alpha \right)|-|\text{log}2\text{FC}\left(S_{C}\right)|$$ (12)

$${\mathrm{BI}}_{\text{Nuc}}=|\text{log}2\text{FC}\left(\alpha \right)|-|\text{log}2\text{FC}\left(S_{T}\right)|$$ (13)

where the log2FC function is defined as $\text{log}2\text{FC}\left(x\right)=\text{log}2\left(x_{\text{perturbation}}\right)-\text{log}2\left(x_{\text{WT}}\right)$. These indices quantify the extent to which a gene can buffer transcriptional perturbations to maintain homeostatic mRNA concentrations in the nucleus, cytoplasm, and whole cell, respectively. Note that a large BI value indicates that changes in transcription (regardless of the direction) do not lead to commensurable changes in mRNA accumulation. The *BI* value also increases with the magnitude of the transcriptional perturbation being buffered. Using RNA accumulation FC alone as a measure of buffering would fail to distinguish between genes whose accumulation levels remain unchanged because they are insensitive to the particular transcriptional perturbation, and those whose levels are maintained due to transcriptional changes compensated by posttranscriptional regulation.

### Model extension and limitations

The model proposed above assumes that nuclear degradation is negligible compared to nuclear export. Increasing evidence supports this assumption. Studies in unperturbed mESCs have shown that, for most of the genes, nuclear retention is primarily driven by export rather than degradation (*31*). However, this may not hold true under transcriptional perturbation. Here, we discuss the potential consequences of explicitly considering nuclear degradation in the interpretation of our results. The steady-state solution of this extended model for nuclear unspliced and spliced RNA as well as cytoplasmic spliced RNA is

$$u_{N}=\frac{\alpha }{\beta }$$ (14)

$$s_{N}=\frac{\alpha }{\eta +\gamma_{N}}$$ (15)

$$s_{C}=\frac{\alpha }{\gamma_{C}}\frac{\eta }{\eta +\gamma_{N}}$$ (16)

Notice that if the condition $\eta \gg \gamma_{N}$ holds true, then the previous results are recovered. In the general case, this model has more parameters than observations and therefore cannot be fully determined. However, we can still draw some interesting conclusions. First, it is straightforward to express the log2 FC in nucleo-cytoplasmic ratio (NCR), i.e., $\text{log}2\left[\left({S_{N}}^{\Delta }/{S_{C}}^{\Delta }\right)/\left({S_{N}}^{\text{WT}}/{S_{C}}^{\text{WT}}\right)\right]$, as the difference between the log2 FC in export and the log2 FC in cytoplasmic degradation

$$\text{log}2\text{FC}\left(\text{NCR}\right)=\text{log}2\text{FC}\left(\eta \right)-\text{log}2\text{FC}\left(\gamma_{C}\right)$$ (17)

This expression indicates that if NCR does not change significantly, changes in nuclear export must be compensated by corresponding changes in cytoplasmic RNA degradation, regardless of the behavior of nuclear degradation. In our case, we show that most of the genes do not show a significant change in NCR upon TIP60 depletion (Fig. 3H).

Furthermore, for genes that nuclear accumulation remains approximately constant, i.e., $\text{log}2\text{FC}\left(S_{N}\right)≃0$, the log2 FC in the transcription rate can be written as

$$\text{log}2\text{FC}\left(\alpha \right)=\text{log}2\text{FC}\left(\eta \right)+\text{lo}g_{2}\left(1+{\gamma_{N}}^{\Delta }/\eta^{\Delta }\right)$$ (18)

Here, we assumed $\eta^{\text{WT}}\gg {\gamma_{N}}^{\text{WT}}$, which, as mentioned previously, has been shown to hold true for most of the genes in mESCs. Thus, changes in transcription may be compensated by changes in export, as concluded from our simpler model earlier. An additional term appears, $\text{lo}g_{2}\left(1+{\gamma_{N}}^{\Delta }/\eta^{\Delta }\right)$, which could account for buffering in the nucleus. However, note that this term is always positive and, therefore, cannot explain the buffering of genes with decreased transcription rates, which constitute the largest fraction upon TIP60 or ATAC depletion.

### Bright-field microscopy

Images were acquired on a ZEISS Axio Observer.Z1/7 bright-field microscope with a Plan-Apochromat 63× objective using ZEN 3.3 (blue edition) acquisition software.

### Immunofluorescence with poly(A) RNA FISH

Coverslips were coated at 37°C with 0.1% gelatin (Sigma-Aldrich, catalog no. G1890) for 1 hour. Cells treated with 1 mM Auxin or DMSO were plated on the precoated coverslips and grown for 24 hours, and cells treated with 500 nM triptolide, 500 nM flavopiridol, or DMSO were grown for 2 hours. Cells were fixed with 4% paraformaldehyde (PFA) (Electron Microscopy Sciences, 15710) in PBS for 15 min, followed by three PBS washes. Permeabilization of cells was performed using 0.5% Triton X-100 (Sigma-Aldrich, X100) in PBS for 10 min, followed by three PBS washes. Cells were blocked with 5% BSA (MP Biomedicals, catalog no. 160069) for 1 hour at room temperature. Cells were then incubated with the primary antibody (anti SC-35/SRRM2, ab11826, Abcam) at a concentration of 1:200 overnight in PBS at 4°C. Cells were washed with PBS three times, followed by incubation with secondary antibodies at a concentration of 1:500 in PBS for 1 hour. After washing twice with PBS, cells were fixed using 4% PFA in PBS for 10 min. Poly(A) RNA FISH was performed as described (*58*). Briefly, after two washes of PBS, cells were incubated with hybridization buffer (15% formamide from Sigma-Aldrich in 1× Saline-Sodium Citrate buffer) for 15 min and then overnight at 37°C in the hybridization buffer containing 1 μM of the poly(A) probe for 100 μl of the final volume, tRNA (0.34 mg/ml), 2 mM Vanadyl Ribonucleoside Complex (Sigma-Aldrich), RNase-free BSA (0.2 mg/ml; Molecular Biology Grade), and 10% dextran sulfate (Sigma-Aldrich, catalog no. D8906). The next day, samples were washed twice for 30 min in the hybridization buffer at 37°C. The nuclei were stained in 4′,6-diamidino-2-phenylindole (DAPI) (1 mg/ml) for 60 min. Cells were then washed once in PBS, followed by mounting the coverslip onto glass slides with ProLong Gold (Invitrogen, P36934). Images were acquired on a Leica confocal microscope with a 63× objective using LAS X acquisition software (Leica).

### Image analysis

Images derived from a single confocal Z-slice were subjected to semi-automated post-processing using Fiji software and a custom macro. Nuclei were initially segmented by applying the Otsu method to the DAPI channel (after applying a Gaussian blur with sigma = 0.5), resulting in a mask outlining nuclear contours. The whole microcolony was subsequently segmented by thresholding the poly(A) + RNA channel (after applying a Gaussian blur with sigma = 3) using the Li method. Nuclear mRNA speckles were identified by thresholding the poly(A) + RNA channel using the MaxEntropy method. A Gaussian blur (sigma = 1) was applied to the resulting mask, followed by a secondary threshold using the Otsu method to generate a mask specific to RNA particles. Particles outside the nuclear contours were excluded, and size and circularity filters were applied to eliminate artifacts. A cytoplasmic mask for the entire microcolony was generated by combining the nuclear mask and the microcolony mask using the XOR logical operation. Visual assessment was used during data processing to ensure accurate nuclear and foci segmentation, with specific measurements being discarded if the semi-automated segmentation method exhibited poor performance, such as inaccurate delineation of nuclear contours or failure to resolve distinct mRNA foci within nuclei.

The mean fluorescence intensity within these contours was then quantified in the poly(A) + RNA channel for each microcolony. For each biological replicate, the area and mean poly(A) fluorescence values of nuclei, as well as the area of mRNA speckles, were normalized by the median value of the untreated condition (DMSO), yielding fold change values. Poly(A) fluorescence values of mRNA speckles were normalized sequentially: first by the average fluorescence of each mESC microcolony and then by the median value of the untreated condition (DMSO) to account for batch effects. These values were pooled together for subsequent statistical analysis. The pooled data underwent a Kruskal-Wallis test followed by Dunn’s multiple comparisons test (comparing DMSO versus triptolide and DMSO versus flavopiridol) or a Mann-Whitney test (comparing DMSO versus auxin). Statistical analysis was performed using GraphPad Prism.

## References

[1] T. Baptista, S. Grünberg, N. Minoungou, M. J. E. Koster, H. T. M. Timmers, S. Hahn, D. Devys, L. Tora, SAGA is a general cofactor for RNA polymerase II transcription. Mol. Cell 68, 130–143.e5 (2017). 28918903 10.1016/j.molcel.2017.08.016 PMC5632562

[2] M. Sun, B. Schwalb, D. Schulz, N. Pirkl, S. Etzold, L. Larivière, K. C. Maier, M. Seizl, A. Tresch, P. Cramer, Comparative dynamic transcriptome analysis (cDTA) reveals mutual feedback between mRNA synthesis and degradation. Genome Res. 22, 1350–1359 (2012). 22466169 10.1101/gr.130161.111 PMC3396375

[3] L. Warfield, S. Ramachandran, T. Baptista, D. Devys, L. Tora, S. Hahn, Transcription of nearly all yeast RNA polymerase II-transcribed genes is dependent on transcription factor TFIID. Mol. Cell 68, 118–129.e5 (2017). 28918900 10.1016/j.molcel.2017.08.014 PMC5679267

[4] S. Berry, M. Müller, A. Rai, L. Pelkmans, Feedback from nuclear RNA on transcription promotes robust RNA concentration homeostasis in human cells. Cell Syst. 13, 454–470.e15 (2022). 35613616 10.1016/j.cels.2022.04.005

[5] K. Helenius, Y. Yang, T. V. Tselykh, H. K. J. Pessa, M. J. Frilander, T. P. Mäkelä, Requirement of TFIIH kinase subunit Mat1 for RNA Pol II C-terminal domain Ser5 phosphorylation, transcription and mRNA turnover. Nucleic Acids Res. 39, 5025–5035 (2011). 21385826 10.1093/nar/gkr107 PMC3130277

[6] M. Sun, B. Schwalb, N. Pirkl, K. C. Maier, A. Schenk, H. Failmezger, A. Tresch, P. Cramer, Global analysis of eukaryotic mRNA degradation reveals Xrn1-dependent buffering of transcript levels. Mol. Cell 52, 52–62 (2013). 24119399 10.1016/j.molcel.2013.09.010

[7] G. Haimovich, D. A. Medina, S. Z. Causse, M. Garber, G. Millán-Zambrano, O. Barkai, S. Chávez, J. E. Pérez-Ortín, X. Darzacq, M. Choder, Gene expression is circular: Factors for mRNA degradation also foster mRNA synthesis. Cell 153, 1000–1011 (2013). 23706738 10.1016/j.cell.2013.05.012

[8] H. T. M. Timmers, L. Tora, Transcript buffering: A balancing act between mRNA synthesis and mRNA degradation. Mol. Cell 72, 10–17 (2018). 30290147 10.1016/j.molcel.2018.08.023

[9] E. Hartenian, B. A. Glaunsinger, Feedback to the central dogma: Cytoplasmic mRNA decay and transcription are interdependent processes. Crit. Rev. Biochem. Mol. Biol. 54, 385–398 (2019). 31656086 10.1080/10409238.2019.1679083 PMC6871655

[10] S. Berry, L. Pelkmans, Mechanisms of cellular mRNA transcript homeostasis. Trends Cell Biol. 32, 655–668 (2022). 35660047 10.1016/j.tcb.2022.05.003

[11] D. C. Rodrigues, M. Mufteev, K. E. Yuki, A. Narula, W. Wei, A. Piekna, J. Liu, P. Pasceri, O. S. Rissland, M. D. Wilson, J. Ellis, Buffering of transcription rate by mRNA half-life is a conserved feature of Rett syndrome models. Nat. Commun. 14, 1896 (2023). 37019888 10.1038/s41467-023-37339-6 PMC10076348

[12] Y. Doyon, J. Côté, The highly conserved and multifunctional NuA4 HAT complex. Curr. Opin. Genet. Dev. 14, 147–154 (2004). 15196461 10.1016/j.gde.2004.02.009

[13] M. Squatrito, C. Gorrini, B. Amati, Tip60 in DNA damage response and growth control: Many tricks in one HAT. Trends Cell Biol. 16, 433–442 (2006). 16904321 10.1016/j.tcb.2006.07.007

[14] T. G. Fazzio, J. T. Huff, B. Panning, An RNAi screen of chromatin proteins identifies Tip60-p400 as a regulator of embryonic stem cell identity. Cell 134, 162–174 (2008). 18614019 10.1016/j.cell.2008.05.031 PMC4308735

[15] M. Shvedunova, A. Akhtar, Modulation of cellular processes by histone and non-histone protein acetylation. Nat. Rev. Mol. Cell Biol. 23, 329–349 (2022). 35042977 10.1038/s41580-021-00441-y

[16] P. B. Chen, J.-H. Hung, T. L. Hickman, A. H. Coles, J. F. Carey, Z. Weng, F. Chu, T. G. Fazzio, Hdac6 regulates Tip60-p400 function in stem cells. eLife 2, e01557 (2013). 24302573 10.7554/eLife.01557 PMC3843111

[17] M. Gomar-Alba, V. Pozharskaia, B. Cichocki, C. Schaal, A. Kumar, B. Jacquel, G. Charvin, J. C. Igual, M. Mendoza, Nuclear pore complex acetylation regulates mRNA export and cell cycle commitment in budding yeast. EMBO J. 41, e110271 (2022). 35735140 10.15252/embj.2021110271 PMC9340480

[18] P. B. Chen, H. V. Chen, D. Acharya, O. J. Rando, T. G. Fazzio, R loops regulate promoter-proximal chromatin architecture and cellular differentiation. Nat. Struct. Mol. Biol. 22, 999–1007 (2015). 26551076 10.1038/nsmb.3122 PMC4677832

[19] A. Bhatnagar, K. Krick, B. C. Karisetty, E. M. Armour, E. A. Heller, F. Elefant, Tip60’s novel RNA-binding function modulates alternative splicing of pre-mRNA targets implicated in Alzheimer’s disease. J. Neurosci. 43, 2398–2423 (2023). 36849418 10.1523/JNEUROSCI.2331-22.2023 PMC10072303

[20] V. Fischer, D. Plassard, T. Ye, B. Reina-San-Martin, M. Stierle, L. Tora, D. Devys, The related coactivator complexes SAGA and ATAC control embryonic stem cell self-renewal through acetyltransferase-independent mechanisms. Cell Rep. 36, 109598 (2021). 34433046 10.1016/j.celrep.2021.109598 PMC8430043

[21] S. Hastreiter, S. Skylaki, D. Loeffler, A. Reimann, O. Hilsenbeck, P. S. Hoppe, D. L. Coutu, K. D. Kokkaliaris, M. Schwarzfischer, K. Anastassiadis, F. J. Theis, T. Schroeder, Inductive and selective effects of GSK3 and MEK inhibition on nanog heterogeneity in embryonic stem cells. Stem Cell Rep. 11, 58–69 (2018). 10.1016/j.stemcr.2018.04.019 PMC6066909 29779897

[22] B. Schwalb, M. Michel, B. Zacher, K. Frühauf, C. Demel, A. Tresch, J. Gagneur, P. Cramer, TT-seq maps the human transient transcriptome. Science 352, 1225–1228 (2016). 27257258 10.1126/science.aad9841

[23] D. Acharya, S. J. Hainer, Y. Yoon, F. Wang, I. Bach, J. A. Rivera-Pérez, T. G. Fazzio, KAT-independent gene regulation by Tip60 promotes ESC self-renewal but not pluripotency. Cell Rep. 19, 671–679 (2017). 28445719 10.1016/j.celrep.2017.04.001 PMC5484067

[24] S. Ravens, C. Yu, T. Ye, M. Stierle, L. Tora, Tip60 complex binds to active Pol II promoters and a subset of enhancers and co-regulates the c-Myc network in mouse embryonic stem cells. Epigenetics Chromatin 8, 45 (2015). 26550034 10.1186/s13072-015-0039-z PMC4636812

[25] E. S. Lee, E. J. Wolf, S. S. J. Ihn, H. W. Smith, A. Emili, A. F. Palazzo, TPR is required for the efficient nuclear export of mRNAs and lncRNAs from short and intron-poor genes. Nucleic Acids Res. 48, 11645–11663 (2020). 33091126 10.1093/nar/gkaa919 PMC7672458

[26] D. Gaidatzis, L. Burger, M. Florescu, M. B. Stadler, Analysis of intronic and exonic reads in RNA-seq data characterizes transcriptional and post-transcriptional regulation. Nat. Biotechnol. 33, 722–729 (2015). 26098447 10.1038/nbt.3269

[27] G. La Manno, R. Soldatov, A. Zeisel, E. Braun, H. Hochgerner, V. Petukhov, K. Lidschreiber, M. E. Kastriti, P. Lönnerberg, A. Furlan, J. Fan, L. E. Borm, Z. Liu, D. van Bruggen, J. Guo, X. He, R. Barker, E. Sundström, G. Castelo-Branco, P. Cramer, I. Adameyko, S. Linnarsson, P. V. Kharchenko, RNA velocity of single cells. Nature 560, 494–498 (2018). 30089906 10.1038/s41586-018-0414-6 PMC6130801

[28] B. Schwanhäusser, D. Busse, N. Li, G. Dittmar, J. Schuchhardt, J. Wolf, W. Chen, M. Selbach, Global quantification of mammalian gene expression control. Nature 473, 337–342 (2011). 21593866 10.1038/nature10098

[29] L. V. Sharova, A. A. Sharov, T. Nedorezov, Y. Piao, N. Shaik, M. S. H. Ko, Database for mRNA half-life of 19 977 genes obtained by DNA microarray analysis of pluripotent and differentiating mouse embryonic stem cells. DNA Res. 16, 45–58 (2009). 19001483 10.1093/dnares/dsn030 PMC2644350

[30] R. Ietswaart, B. M. Smalec, A. Xu, K. Choquet, E. McShane, Z. M. Jowhar, C. K. Guegler, A. R. Baxter-Koenigs, E. R. West, B. X. H. Fu, L. Gilbert, S. N. Floor, L. S. Churchman, Genome-wide quantification of RNA flow across subcellular compartments reveals determinants of the mammalian transcript life cycle. Mol. Cell 84, 2765–2784.e16 (2024). 38964322 10.1016/j.molcel.2024.06.008 PMC11315470

[31] D. Steinbrecht, I. Minia, M. Milek, J. Meisig, N. Blüthgen, M. Landthaler, Subcellular mRNA kinetic modeling reveals nuclear retention as rate-limiting. Mol. Syst. Biol. 20, 1346–1371 (2024). 39548324 10.1038/s44320-024-00073-2 PMC11611909

[32] J. M. Müller, K. Moos, T. Baar, K. C. Maier, K. Zumer, A. Tresch, Nuclear export is a limiting factor in eukaryotic mRNA metabolism. PLOS Comput. Biol. 20, e1012059 (2024). 38753883 10.1371/journal.pcbi.1012059 PMC11135743

[33] L. Herzel, D. S. M. Ottoz, T. Alpert, K. M. Neugebauer, Splicing and transcription touch base: Co-transcriptional spliceosome assembly and function. Nat. Rev. Mol. Cell Biol. 18, 637–650 (2017). 28792005 10.1038/nrm.2017.63 PMC5928008

[34] F. Ding, M. B. Elowitz, Constitutive splicing and economies of scale in gene expression. Nat. Struct. Mol. Biol. 26, 424–432 (2019). 31133700 10.1038/s41594-019-0226-x PMC6663491

[35] D. E. Sterner, S. L. Berger, Acetylation of histones and transcription-related factors. Microbiol. Mol. Biol. Rev. 64, 435–459 (2000). 10839822 10.1128/mmbr.64.2.435-459.2000 PMC98999

[36] V. Sapountzi, I. R. Logan, C. N. Robson, Cellular functions of TIP60. Int. J. Biochem. Cell Biol. 38, 1496–1509 (2006). 16698308 10.1016/j.biocel.2006.03.003

[37] H. Xiao, J. Chung, H.-Y. Kao, Y.-C. Yang, Tip60 is a co-repressor for STAT3. J. Biol. Chem. 278, 11197–11204 (2003). 12551922 10.1074/jbc.M210816200

[38] V. Aksenova, A. Smith, H. Lee, P. Bhat, C. Esnault, S. Chen, J. Iben, R. Kaufhold, K. C. Yau, C. Echeverria, B. Fontoura, A. Arnaoutov, M. Dasso, Nucleoporin TPR is an integral component of the TREX-2 mRNA export pathway. Nat. Commun. 11, 4577 (2020). 32917881 10.1038/s41467-020-18266-2 PMC7486939

[39] S. Shibata, Y. Matsuoka, Y. Yoneda, Nucleocytoplasmic transport of proteins and poly(A)^+^ RNA in reconstituted Tpr-less nuclei in living mammalian cells. Genes Cells 7, 421–434 (2002). 11952838 10.1046/j.1365-2443.2002.00525.x

[40] K. Tokunaga, T. Shibuya, Y. Ishihama, H. Tadakuma, M. Ide, M. Yoshida, T. Funatsu, Y. Ohshima, T. Tani, Nucleocytoplasmic transport of fluorescent mRNA in living mammalian cells: Nuclear mRNA export is coupled to ongoing gene transcription. Genes Cells 11, 305–317 (2006). 16483318 10.1111/j.1365-2443.2006.00936.x

[41] M. Dori-Bachash, O. Shalem, Y. S. Manor, Y. Pilpel, I. Tirosh, Widespread promoter-mediated coordination of transcription and mRNA degradation. Genome Biol. 13, R114 (2012). 23237624 10.1186/gb-2012-13-12-r114 PMC4056365

[42] B. Slobodin, A. Bahat, U. Sehrawat, S. Becker-Herman, B. Zuckerman, A. N. Weiss, R. Han, R. Elkon, R. Agami, I. Ulitsky, I. Shachar, R. Dikstein, Transcription dynamics regulate poly(A) tails and expression of the RNA degradation machinery to balance mRNA levels. Mol. Cell 78, 434–444.e5 (2020). 32294471 10.1016/j.molcel.2020.03.022

[43] A. Gallego, J. M. Fernández-Justel, S. Martín-Vírgala, M. M. Maslon, M. Gómez, Slow RNAPII transcription elongation rate, low levels of RNAPII pausing, and elevated histone H1 content at promoters associate with higher m6A deposition on nascent mRNAs. Genes 13, 1652 (2022). 36140819 10.3390/genes13091652 PMC9498810

[44] Y. Lee, J. Choe, O. H. Park, Y. K. Kim, Molecular mechanisms driving mRNA degradation by m6A modification. Trends Genet. 36, 177–188 (2020). 31964509 10.1016/j.tig.2019.12.007

[45] B. P. Kleinstiver, V. Pattanayak, M. S. Prew, S. Q. Tsai, N. T. Nguyen, Z. Zheng, J. K. Joung, High-fidelity CRISPR-Cas9 nucleases with no detectable genome-wide off-target effects. Nature 529, 490–495 (2016). 26735016 10.1038/nature16526 PMC4851738

[46] C. Engler, R. Gruetzner, R. Kandzia, S. Marillonnet, Golden gate shuffling: A one-pot DNA shuffling method based on type IIs restriction enzymes. PLOS ONE 4, e5553 (2009). 19436741 10.1371/journal.pone.0005553 PMC2677662

[47] B. Rädle, A. J. Rutkowski, Z. Ruzsics, C. C. Friedel, U. H. Koszinowski, L. Dölken, Metabolic labeling of newly transcribed RNA for high resolution gene expression profiling of RNA synthesis, processing and decay in cell culture. J. Vis. Exp. 78, 50195 (2013). 10.3791/50195 PMC3854562 23963265

[48] M. Rabani, J. Z. Levin, L. Fan, X. Adiconis, R. Raychowdhury, M. Garber, A. Gnirke, C. Nusbaum, N. Hacohen, N. Friedman, I. Amit, A. Regev, Metabolic labeling of RNA uncovers principles of RNA production and degradation dynamics in mammalian cells. Nat. Biotechnol. 29, 436–442 (2011). 21516085 10.1038/nbt.1861 PMC3114636

[49] L. Dölken, Z. Ruzsics, B. Rädle, C. C. Friedel, R. Zimmer, J. Mages, R. Hoffmann, P. Dickinson, T. Forster, P. Ghazal, U. H. Koszinowski, High-resolution gene expression profiling for simultaneous kinetic parameter analysis of RNA synthesis and decay. RNA 14, 1959–1972 (2008). 18658122 10.1261/rna.1136108 PMC2525961

[50] L. Wachutka, L. Caizzi, J. Gagneur, P. Cramer, Global donor and acceptor splicing site kinetics in human cells. eLife 8, e45056 (2019). 31025937 10.7554/eLife.45056 PMC6548502

[51] M. W. Pfaffl, A new mathematical model for relative quantification in real-time RT-PCR. Nucleic Acids Res. 29, e45 (2001). 11328886 10.1093/nar/29.9.e45 PMC55695

[52] M. Martin, Cutadapt removes adapter sequences from high-throughput sequencing reads. EMBnet. J 17, 10–12 (2011).

[53] A. Dobin, C. A. Davis, F. Schlesinger, J. Drenkow, C. Zaleski, S. Jha, P. Batut, M. Chaisson, T. R. Gingeras, STAR: Ultrafast universal RNA-seq aligner. Bioinformatics 29, 15–21 (2013). 23104886 10.1093/bioinformatics/bts635 PMC3530905

[54] S. Anders, P. T. Pyl, W. Huber, HTSeq—A Python framework to work with high-throughput sequencing data. Bioinformatics 31, 166–169 (2015). 25260700 10.1093/bioinformatics/btu638 PMC4287950

[55] M. I. Love, W. Huber, S. Anders, Moderated estimation of fold change and dispersion for RNA-seq data with DESeq2. Genome Biol. 15, 550 (2014). 25516281 10.1186/s13059-014-0550-8 PMC4302049

[56] Y. Benjamini, Y. Hochberg, Controlling the false discovery rate: A practical and powerful approach to multiple testing. J. R. Stat. Soc. 57, 289–300 (1995).

[57] B. M. Smalec, R. Ietswaart, K. Choquet, E. McShane, E. R. West, L. S. Churchman, Genome-wide quantification of RNA flow across subcellular compartments reveals determinants of the mammalian transcript life cycle. bioRxiv 504696 [Preprint] (2022). 10.1101/2022.08.21.504696. PMC11315470 38964322

[58] N. Tsanov, A. Samacoits, R. Chouaib, A.-M. Traboulsi, T. Gostan, C. Weber, C. Zimmer, K. Zibara, T. Walter, M. Peter, E. Bertrand, F. Mueller, smiFISH and FISH-quant–A flexible single RNA detection approach with super-resolution capability. Nucleic Acids Res. 44, e165 (2016). 27599845 10.1093/nar/gkw784 PMC5159540

