# Supplementary material for: Gene-specific transcript buffering revealed by perturbation of coactivator complexes
Source: Sci Adv. 2025 Mar 19;11(12):eadr1492. doi: 10.1126/sciadv.adr1492 (PMC11922027; doi:10.1126/sciadv.adr1492)
Supplement: Supplementary file 1 — Figs. S1 to S12 Table S1 Legends for data S1 to S3 [file sciadv.adr1492_sm.pdf]

Science Advances
AAAS

Supplementary Materials for

Gene-specific transcript buffering revealed by perturbation of coactivator complexes

Faezeh Forouzanfar *et al.*

Corresponding author: Nacho Molina, nacho.molina@igbmc.fr; Manuel Mendoza, manuel.mendoza@igbmc.fr

Sci. Adv. **11**, eadr1492 (2025)
DOI: 10.1126/sciadv.adr1492

The PDF file includes:

Figs. S1 to S12
Table S1
Legends for data S1 to S3

Other Supplementary Material for this manuscript includes the following:

Data S1 to S3

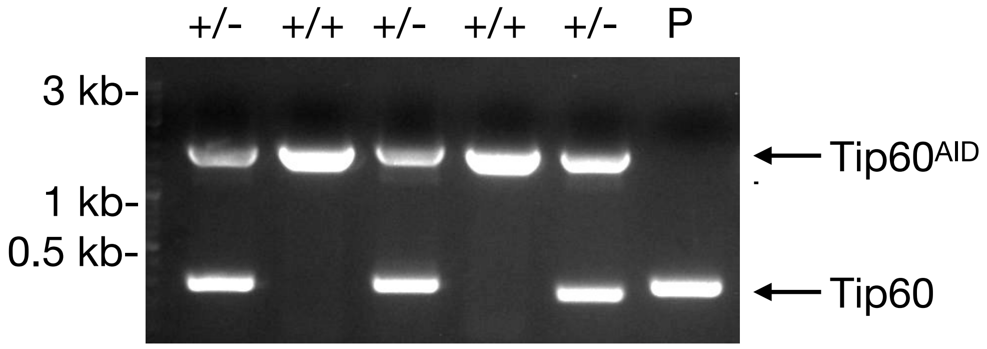

Fig. S1. Genomic integration of the sequence AID-FLAG-BioTagP2A-EGFP into genomic loci of *Kat5* (Tip60) gene. PCR of independent mESC clones.

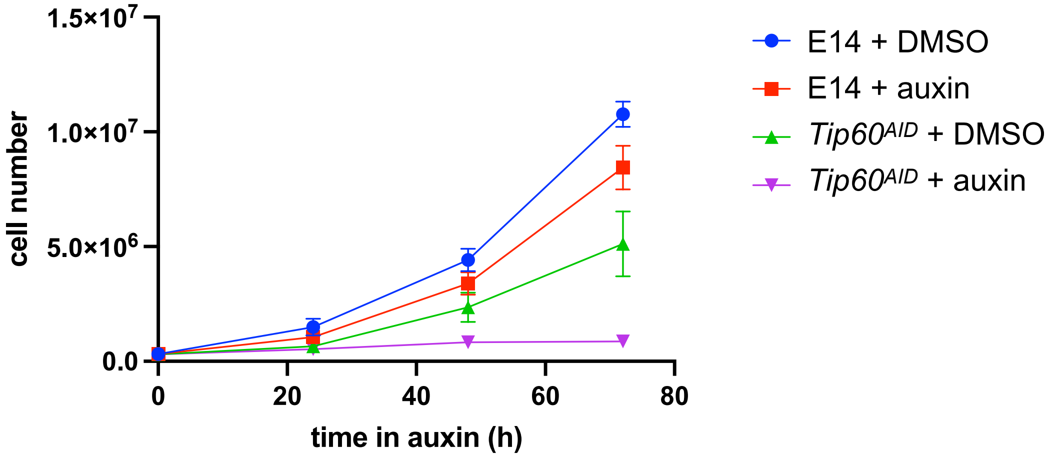

Fig. S2. Cell number of the indicated cells grown in LIF medium and treated with DMSO or 1 mM auxin. Mean and SEM of n=3 independent experiments are shown.

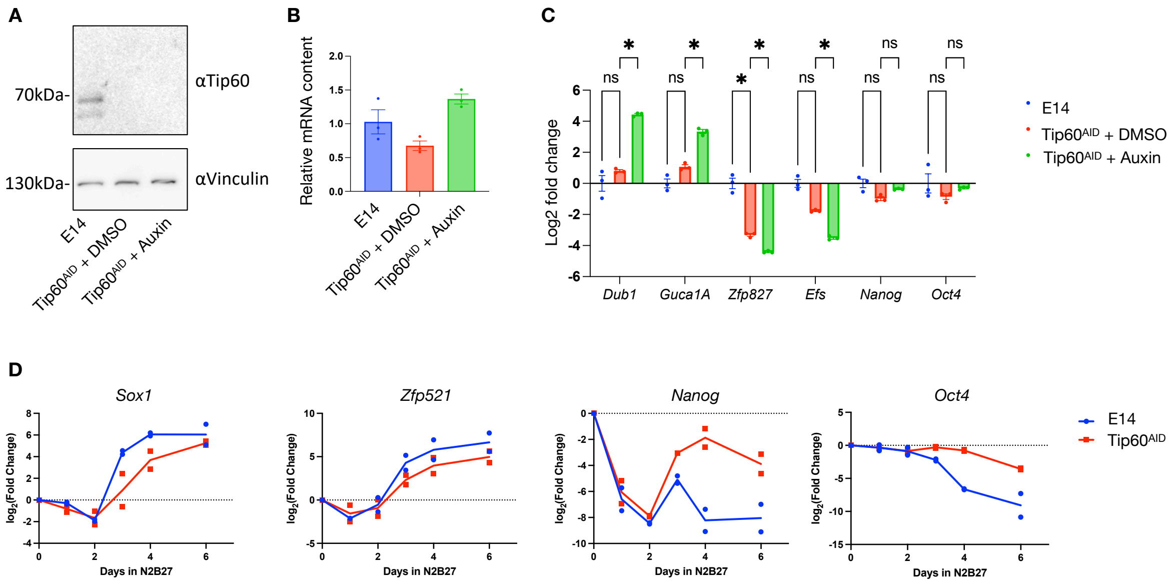

Figure S3. Effects of TIP60-AID tag on Gene Expression and Differentiation. (A) Immunoblot analysis showing TIP60 protein levels in E14 and TIP60-AID ESCs treated with DMSO or auxin for TIP60 depletion. Vinculin serves as a loading control. (B) Quantification of mRNA content using RT-qPCR for TIP60, showing expression levels under DMSO and auxin treatments in E14 and TIP60-AID ESCs. (C) Gene expression analysis (RT-qPCR) of TIP60 target genes (DUB1, GUCA1A, ZFP827, EFS) and pluripotency markers (NANOG, OCT4) in TIP60-AID ESCs treated with DMSO or auxin, compared to control E14 ESCs. Data are presented as log2 fold changes relative to E14 controls. Statistical significance is indicated (*, $p < 0.05$; ns, not significant). (D) Time course of gene expression changes in ESCs during differentiation in N2B27 medium. Expression of SOX1, ZFP521, NANOG, and OCT4 was analyzed in control E14 ESCs and TIP60-AID ESCs. Data are plotted as log2 fold changes relative to day 0. The housekeeping mRNA PPIA was used for normalisation in B-D.

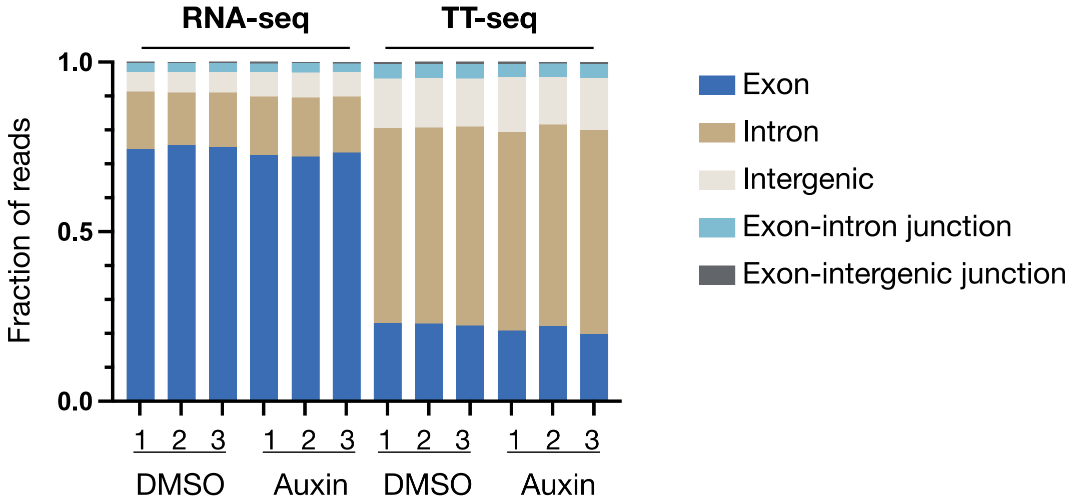

Figure S4. Proportion of reads mapped to the indicated genomic elements of RNA-seq and TT-seq experiments. Three independent replicates are shown (1-3).

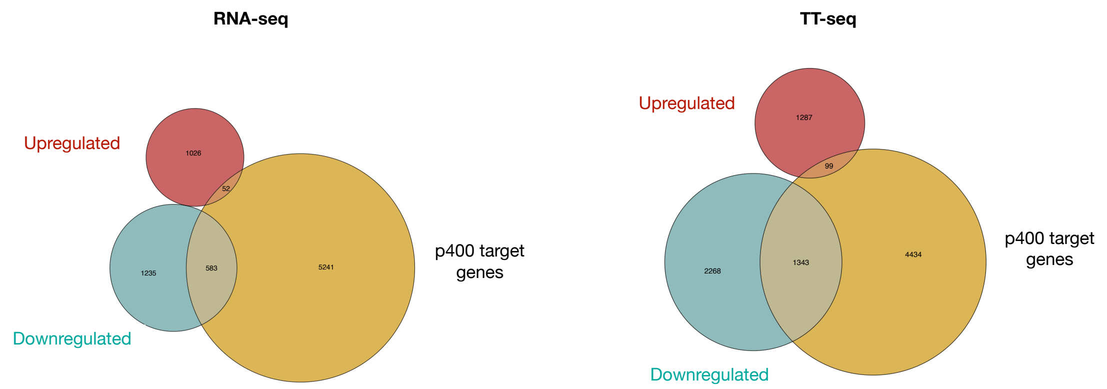

Figure S5. Overlap between differentially expressed genes in DMSO vs auxin-treated *Tip60^{AID}* cells and p400-associated genes (ref. 18) assessed by RNA-seq and TT-seq.

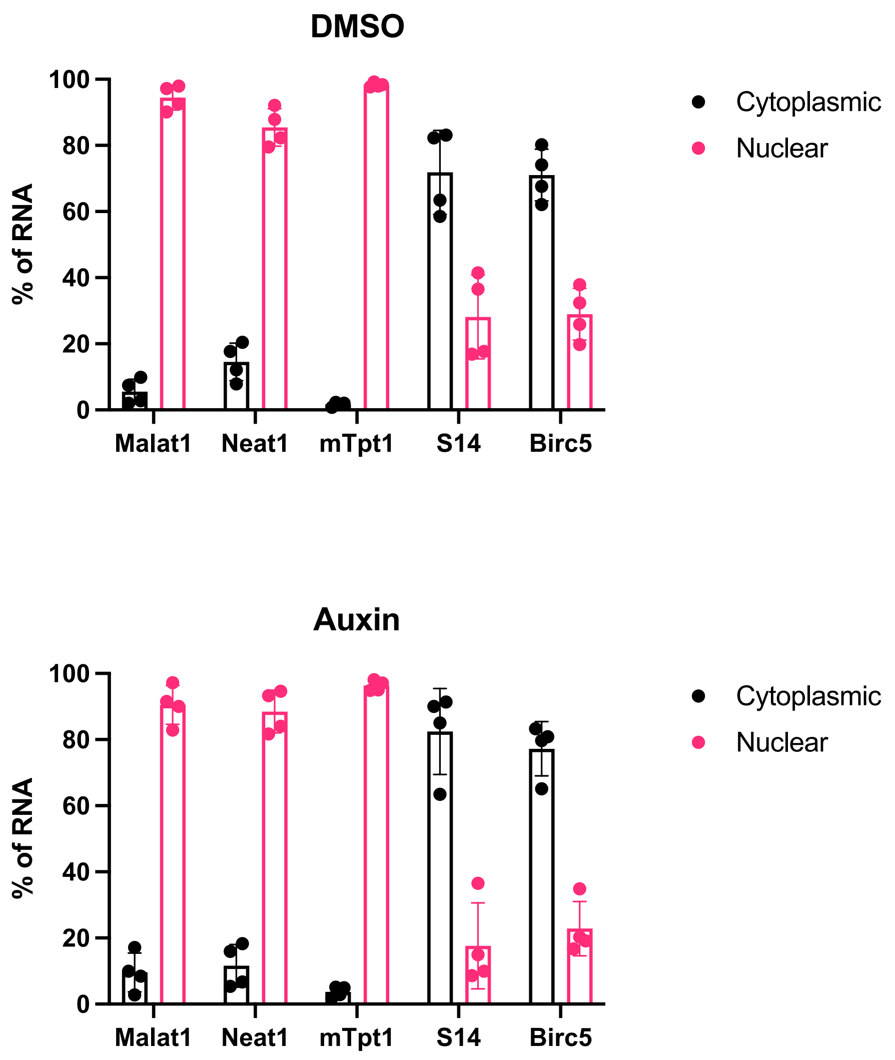

Figure S6. RT-qPCR analysis of the indicated RNAs from nuclear and cytoplasmic fractions. Malat1 and Neat1 are nuclear lncRNAs. mTpt1 corresponds to an intronic region. Both RPS14 (S14) and BIRC5 mRNAs are expected to localise within the cytoplasm. Mean and SD of n=4 biological replicates.

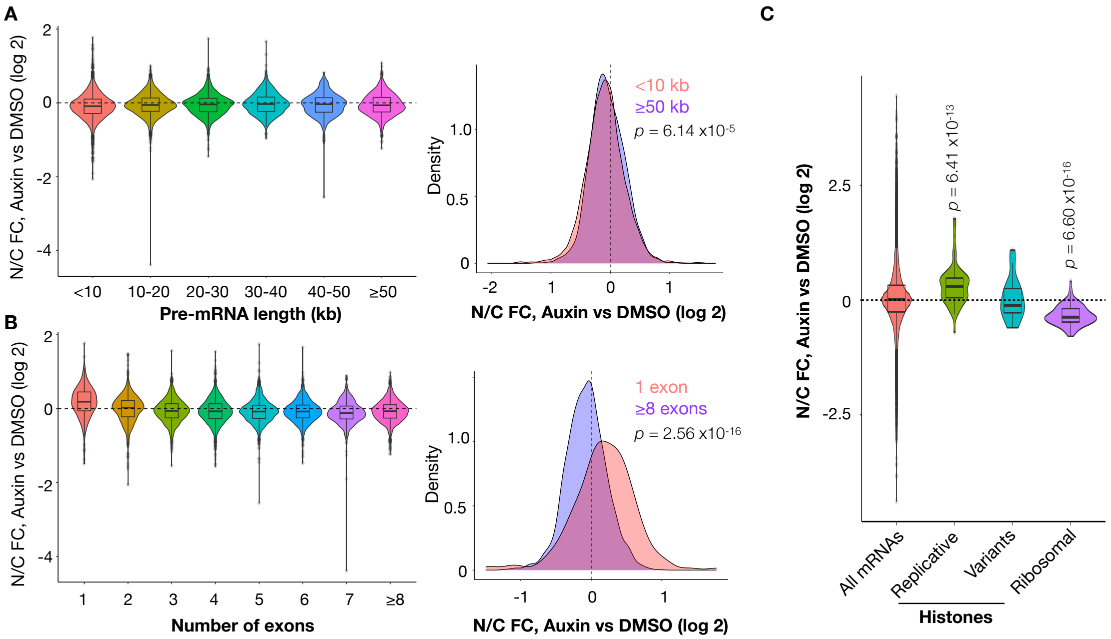

Figure S7: TIP60 depletion affects the N/C ratio of specific RNA categories. A: Violin plot of TIP60-dependent changes in nuclear/cytoplasmic ratio (auxin v DMSO fold change) for pre-mRNAs of varying lengths in 10 kb windows, and density plot showing that very short (<10 kb) and very long (>50 kb) pre-mRNAs are not significantly enriched in the nucleus after Tip60 depletion. B: Violin and density plots as in (A), but for pre-mRNAs containing various numbers of introns. Note that intronless pre-mRNAs show a slightly higher nuclear accumulation after TIP60 depletion. C: Violin plot as in (A-B) for specific mRNA classes. Note that replicative histone mRNAs are slightly enriched in the nucleus of TIP60-depleted cells, whereas ribosomal protein genes are slightly enriched in the cytoplasm.

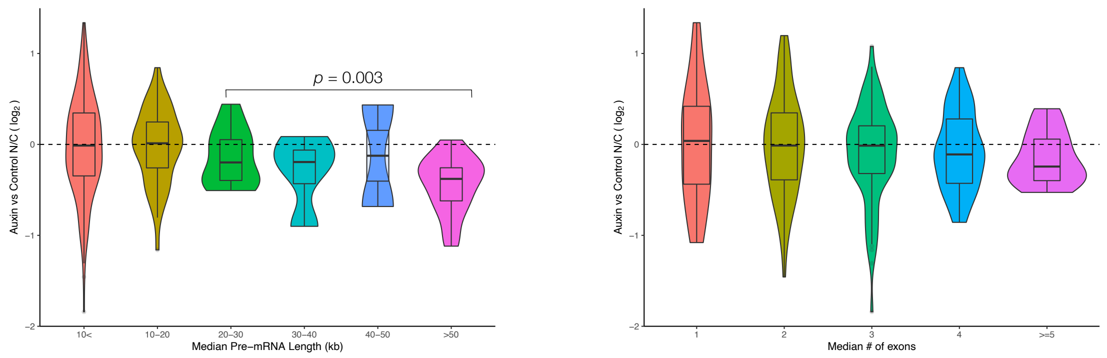

Figure S8. **TIP60-dependent changes in nuclear/cytoplasmic ratio (auxin v DMSO fold change) for lncRNAs of varying lengths and for lncRNAs containing various numbers of introns.** Note that lncRNAs of length >50 kb show a slightly higher cytoplasmic accumulation after TIP60 depletion.

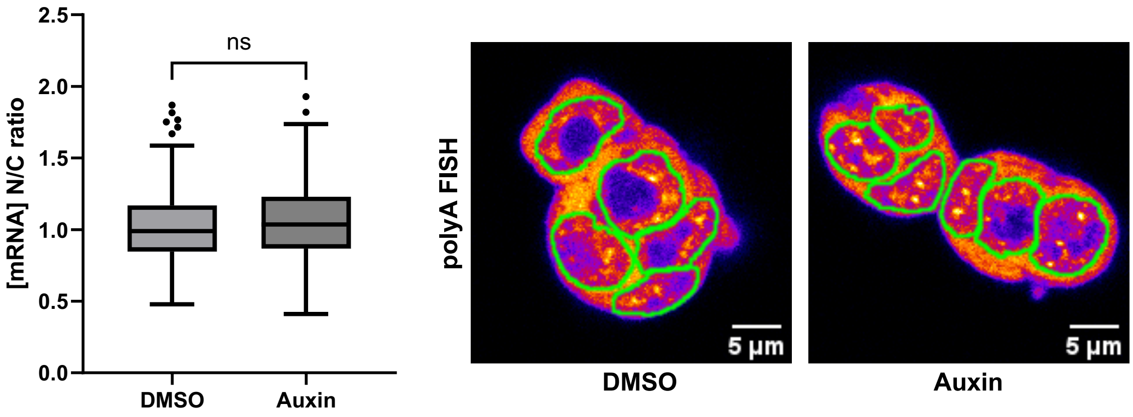

Figure S9. Nuclear-to-cytoplasmic (N/C) mRNA ratios measured using poly(A) RNA FISH in TIP60-AID ESCs treated with DMSO (control) or auxin (TIP60 depletion). Statistical analysis indicates no significant differences between the conditions (ns, not significant).

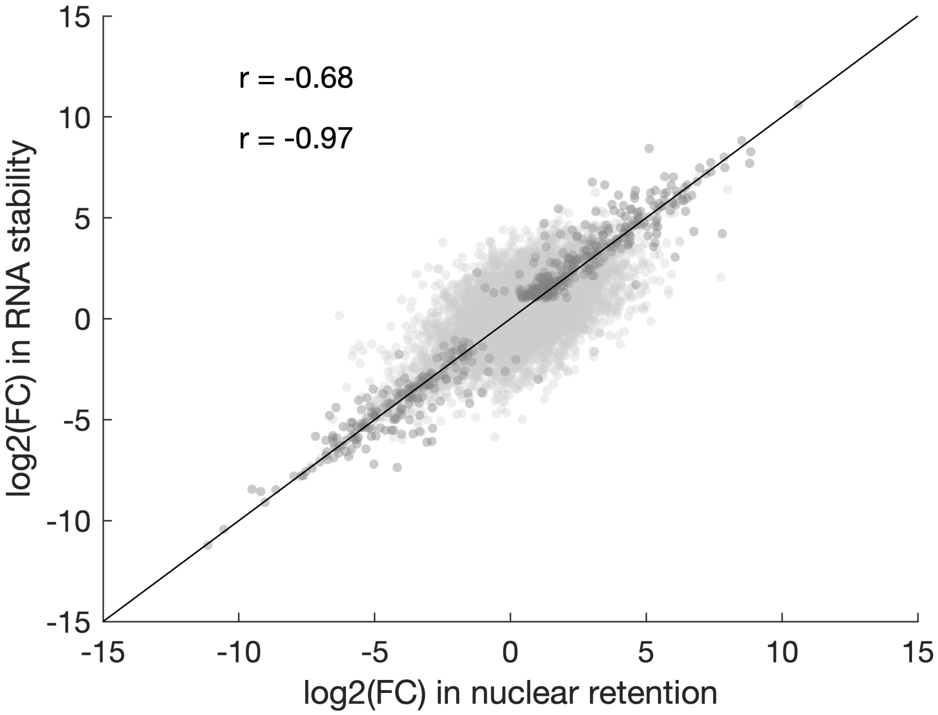

Figure S10. Scatter plot showing the correlation between the fold-change in nuclear retention and the fold change in cytoplasmic stability for RNAs in TIP60 depletion experiments.

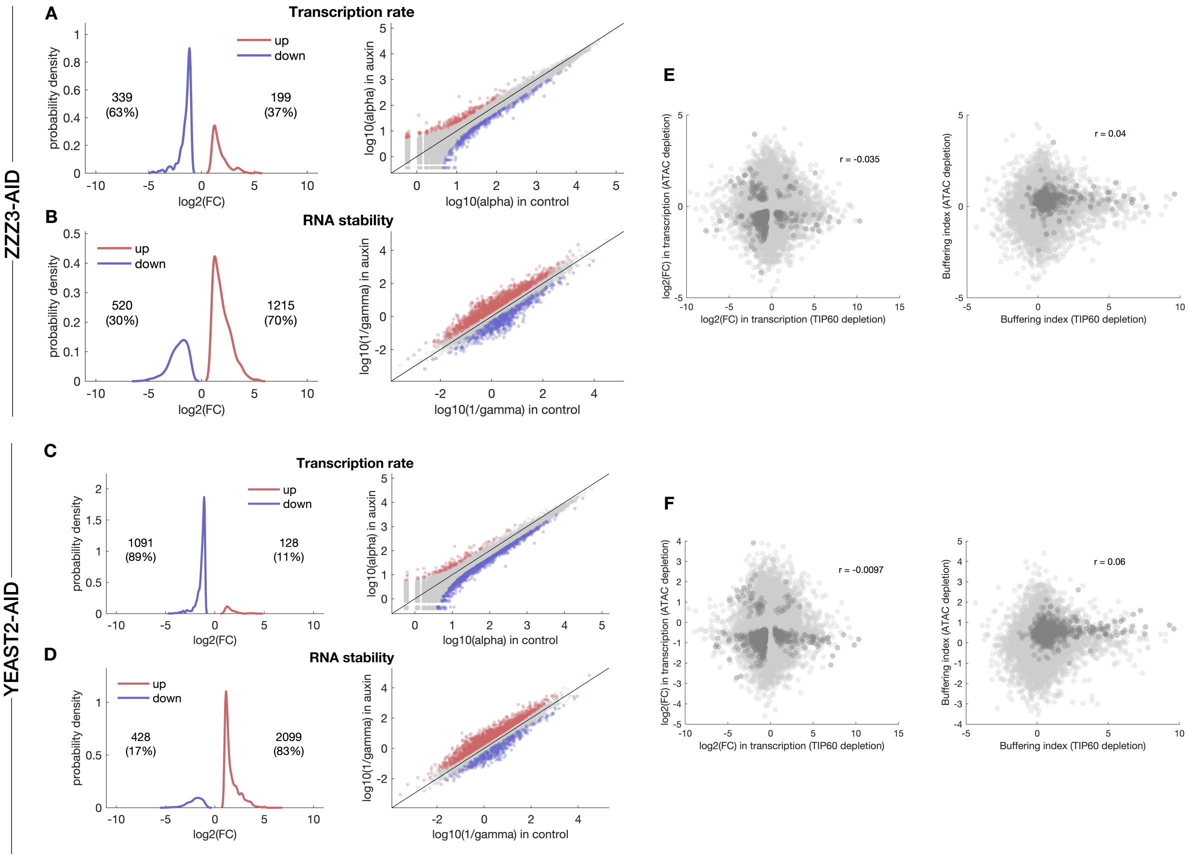

Figure S11. Integration of TT-seq and total RNA-seq of ATAC depletion experiments. (A-B) *Left*: distribution of log2 fold changes (FC) in transcription rate (α) (A) and RNA stability (B) upon Zzz3 depletion. Only genes with a log2 FC bigger than 1 (red) or smaller than -1 (blue) and a p-value < 0.05 were considered. The numbers of genes that pass these criteria are shown. *Right*: scatter plot of gene-specific rates for transcription (A) and RNA stability (B) in control vs. auxin. Blue and red dots represent significant (p-value < 0.01) upregulated genes (log2 FC > 1) and downregulated genes (log2 FC < -1). Gene numbers and relative percentages in each category are indicated. C-D: similar plots as above for the integration of TT-seq and total RNA-seq of YEATS2 depletion experiments. (E) Scatter plots showing lack of correlation between changes in transcription (left) and changes in buffering index (right) of TIP60 depleted and ZZZ3-depleted cells. (F) Scatter plots showing lack of correlation between changes in transcription (left) and changes in buffering index (right) of TIP60 depleted and YEATS2-depleted cells.

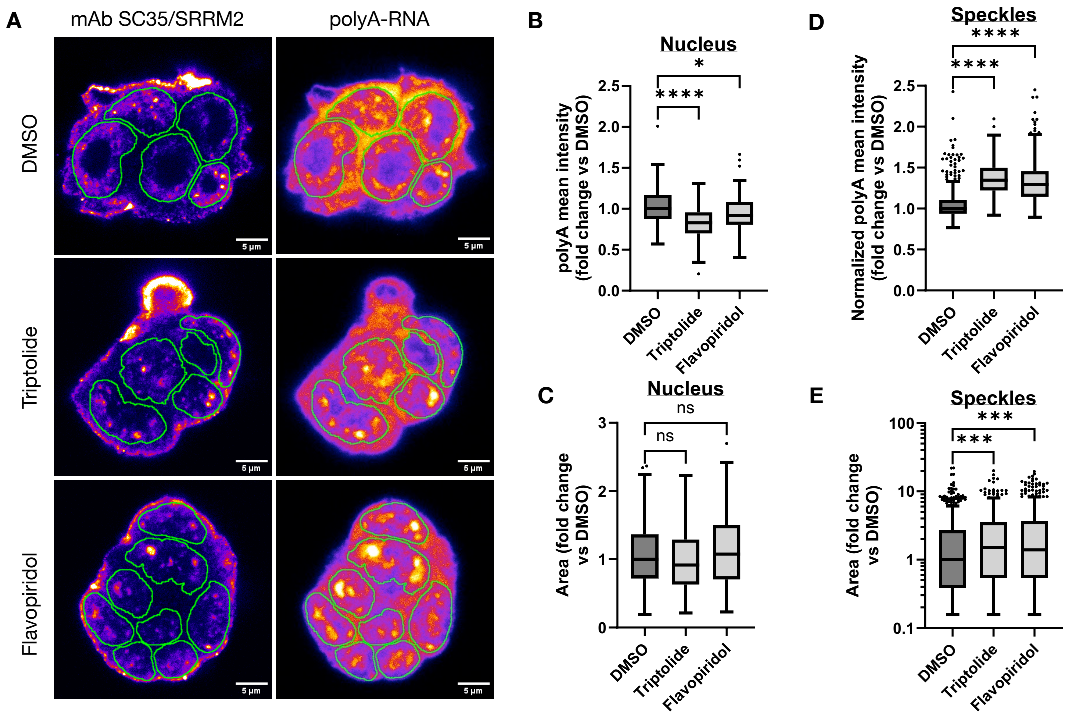

Figure S12: Nuclear redistribution of mRNA upon inhibition of transcription. (A) Representative images of mouse embryonic stem cell colonies treated with DMSO, Triptolide, or Flavopiridol for 2 hours. Cells were fixed and stained with DAPI to label nuclei, SC35/SRRM2 monoclonal antibody labelling nuclear speckles, and Cy3-tagged polyT oligonucleotide labelling mRNA. Nuclear contours are depicted in green. Scale bar, 5 µm. (B-E) Box plots showing the fold change compared to the DMSO condition for the following parameters: (B) nuclear polyA mean intensity, (C) nuclear area, (D) normalised polyA mean intensity on speckles, and (E) speckle area (note the logarithmic scale on the y-axis). Whiskers were calculated using the Tukey method. ns, not significant ($p > 0.05$); * $p \leq 0.05$; ** $p \leq 0.01$; *** $p \leq 0.001$; **** $p \leq 0.0001$. Cells were pooled from two independent experiments.

Table S1. RT- qPCR primers

Gene	Forward primer [5` => 3`]	Reverse primer [5` => 3`]
MALAT1	GGCCAGCTGCAAACATTCAA	TGCAGTGTGCCAATGTTTCG
NEAT1	GTACTGGTGAAGGTGTGGGG	TGTCGAGACAAGTATGCCCG
RPS14	GGAAACCATCTGCCGAGTGA	GTTTGATGTGCAGGGCAGTG
BIRC5	CGCGATTTGAATCCTGCGTT	AGGGCCAGTTCTTGAAGGTG
TPT1	TTAAGCACATCCTTGCTAATTTCA	TGTACGAGACAGCAAACAGACTTT
PPIA	TTACCCATCAAACCATTCCTTCTG	AACCCAAAGAACTTCAGTGAGAGC
OCT4	CCAATCAGCTTGGGCTAGAG	CTGGGAAAGGTGTCCCTGTA
NANOG	TACCTCAGCCTCCAGCAGAT	GCAATGGATGCTGGGATACT
ZFP827	CCGCCTCAGTCCTTGGAATT	GAGATTGTCCTTGCGAGCAC
EFS	GACTTGATGGTTGGTGCCTC	CAGAAGAGGGTGAGCAGGAT
GUCA1A	CCTCAAGGGCAAAGTGGAAC	AATTCCTCGGCACTCATGGA
DUB1	ACACACCACCTCTAGCTGAC	GAAACTCGTGGGCATCTTCC
TIP60	GGGGAGATAATCGAGGGCTG	TCTTGGTAGGTGTCTTGGCC

4sU RNA-seq validation

Gene	Forward primer [5` => 3`]	Reverse primer [5` => 3`]
TPT1	TTAAGCACATCCTTGCTAATTTCA	TGTACGAGACAGCAAACAGACTTT
CFL1	TATGAGACCAAGGAGAGCAAGAA	GTTAAGCTCTGAGAAAGGGAACC

D. melanogaster spike

dRpl12	AAGGGAACCTGCAAGGAAGT	CCCTCGTTCAGTTCGTCAATA
Rp49	GACGCTTCAAGGGACAGTATCTG	AAACGCGGTTCTGCATGAG

Data S1.
Estimated parameters of RNA metabolism and buffering index in TIP60-AID cells treated with DMSO vs Auxin.

Data S2.
Estimated parameters of RNA metabolism and buffering index in ZZZ3-AID cells treated with DMSO vs Auxin.

Data S3.
Estimated parameters of RNA metabolism and buffering index in YEATS2-AID cells treated with DMSO vs Auxin.
